# A Catalyst-Coated Mesoporous Carbon–Membrane Electrode Assembly for In Situ Soft X‑ray XPS and NEXAFS Studies of Electrocatalytic Interfaces

**DOI:** 10.1021/acselectrochem.5c00554

**Published:** 2026-03-17

**Authors:** James J. C. Counter, Santosh Kumar, Christopher M. Zalitis, Mark Clapp, Alexander. I. Large, David C. Grinter, Matthijs A. van Spronsen, Pilar Ferrer, Burcu Karagoz, Tugce Eralp Erden, Roger A. Bennett, Georg Held

**Affiliations:** † Diamond Light Source, Harwell Science and Innovation Campus, Didcot OX11 0DE, U.K.; ‡ Department of Chemistry, 6816 University of Reading, Reading RG6 6DX, U.K.; § Johnson Matthey Technology Centre, Blounts Court Road, Sonning Common, Reading RG4 9NH, U.K.

**Keywords:** operando XPS, NEXAFS, mesoporous carbon, membrane electrode assembly, electrocatalysis, oxygen evolution reaction, soft X-ray spectroscopy, near-ambient pressure, in situ XPS, in situ NEXAFS

## Abstract

In situ soft X-ray spectroscopy provides direct insight into the electronic structure of electrocatalysts under realistic reaction conditions but remains technically challenging due to the need to combine aqueous electrochemistry with ultra-high-vacuum detection. Here, we present a mesoporous carbon–membrane working electrode assembly (WEA) that enables window-free in situ XPS and NEXAFS measurements during electrochemical reactions. The design integrates a Nafion proton-exchange membrane with a mesoporous carbon–ionomer contact layer and a thin IrO_*x*_ catalyst layer, providing continuous electronic and protonic pathways and stable hydration through the membrane. By tuning the chamber water vapor pressure to 8 mbar, the WEA maintains a nanometer-thin water layer sufficient for the oxygen evolution reaction (OER) while preserving photoelectron detection efficiency. A robust peristaltic pump integrated with an alumina-bed water vapor dosing system maintains steady-state hydration at 6–10 mbar with <±0.1 mbar variation, enabling reproducible in situ spectra over extended periods. In situ Ir 4f and O 1s XPS reveal oxidation of Ir^3+^/Ir^4+^ to Ir^4+^/Ir^5+^ and dynamic changes in hydroxyl and lattice oxygen species, while O K-edge NEXAFS identify the formation of potential-stabilized μ_2_–O and μ_1_–O oxygen ligand species at OER. The WEA thus provides a quantitative, window-free platform for probing electrochemical interfaces under near-ambient conditions and establishes a general methodology for in situ soft X-ray studies of functional electrocatalysts, closely resembling the architecture and operation of industrial membrane-based water electrolyzers. This approach establishes a reliable methodology for coupling electrochemistry with the element specific soft X-ray spectroscopy under realistic reaction conditions.

## Graphical abstract

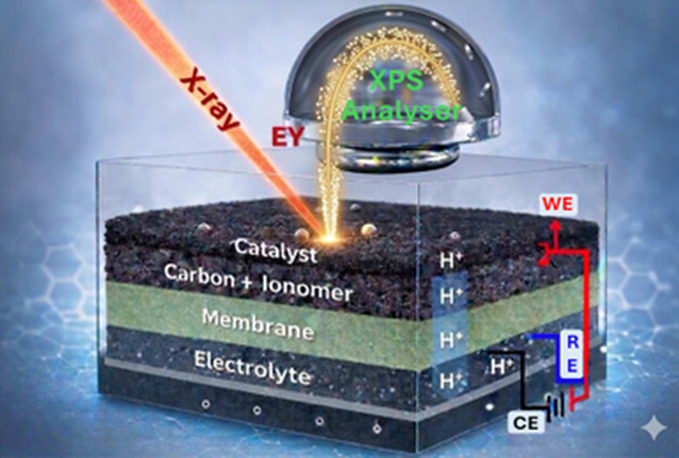

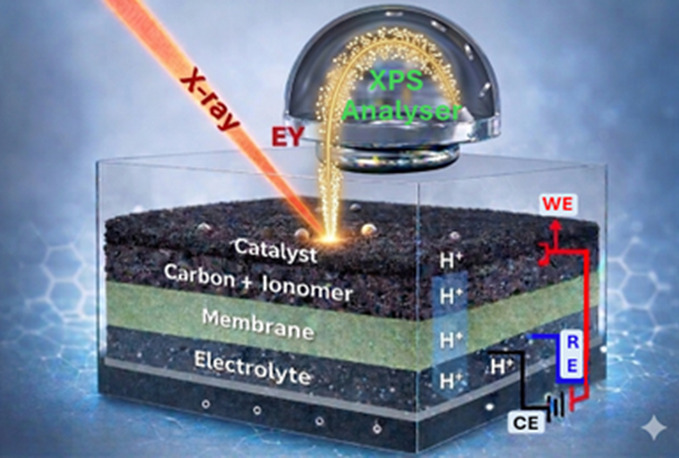

## Introduction

Understanding the structure and reactivity of electrocatalyst surfaces under realistic operating conditions is essential for advancing key electrochemical reactions, such as water electrolysis, CO_2_ electrolysis, and oxygen reduction, in energy conversion technologies.^1–4^ These reactions involve complex charge-transfer and bond-breaking steps at the solid–liquid interface, where the local environment strongly influences activity and stability. Despite major progress in electrochemical characterization, direct observation of the chemical and electronic structure of catalyst surfaces during reaction remains a central challenge.

Soft X-ray techniques including X-ray photoelectron spectroscopy (XPS) and near-edge X-ray absorption fine structure (NEXAFS) offer unique element- and oxidation-state sensitivity to probe catalytic processes at the atomic level.^3,5–13^ In recent years, near-ambient-pressure (NAP) XPS and NEXAFS have extended these capabilities toward realistic reaction environments.^3,5–15^ However, integrating these techniques with electrochemical control introduces fundamental engineering conflicts between the needs of electrochemistry and those of spectroscopy. From the electrochemical perspective, three key conditions must be met: (1) a continuous electronic and ionic pathway between the catalyst and external circuit, ensuring efficient charge transport; (2) effective mass transport to the active sites and product removal (e.g., O_2_) to prevent concentration gradients; and (3) a stable potential reference and current distribution across the working electrode surface. In contrast, the soft X-ray measurement imposes nearly opposite constraints: (1) the catalyst surface must remain in direct or near line-of-sight to the incident X-ray beam and the electron analyzer to maximize signal intensity; and (2) the hydration layer on the surface must be extremely thin; typically only a few nanometers, since even small increases in water thickness exponentially attenuate photoelectrons (escape depth ≈ 3–5 nm for 1000 eV)^4^ ^.^ ^16^ This thin hydration layer must nevertheless be sufficient to maintain electrochemical function and transport ions. Therefore, any in situ or operando electrochemical cell for XPS/NEXAFS represents a compromise: it must sustain simultaneous electrochemical activity and vacuum-compatible soft X-ray detection while stabilizing a nanometer-scale water layer at the interface.

To probe the solid–liquid interface, several experimental strategies have been developed, including static thin liquid layers (where the vapor pressure is sufficiently low),^17–19^ liquid replenishment via a capillary,^6^ vapor condensation “dip-and-pull” techniques,^20–23^ liquid jets,^24–26^ and liquid cells. Among these, liquid-cell configurations,^3,8,27–29^ provide the most realistic and stable environment for spectroscopic interrogation of electrochemical interfaces, closely resembling both laboratory electrochemical systems and practical electrolyzers.

Recently, we developed a liquid-based electrochemical flow cell for in situ NAP-XPS and NAP-NEXAFS studies.^5^ However, the design of the working electrode assembly (WEA) is critical for surface-sensitive in situ and operando techniques such as soft X-ray NAP-XPS and NEXAFS. Catalyst-coated membranes capped with graphene can be employed, as graphene is transparent to both X-rays and photoelectrons while retaining electrolyte contact and electronic conductivity. Nevertheless, graphene layers can complicate the interpretation of spectro-electrochemical data: graphene with defects likely oxidizes, diminishing its conductivity and introducing an additional graphene oxide–electrolyte interface.^30–35^ Moreover, product accumulation between the electrocatalyst and graphene may result in probing the catalyst–product rather than the catalyst–electrolyte interface. When a polymer exchange membrane is used beneath the catalyst, restricted electrolyte transport due to electro-osmotic drag can lead to dehydration and mass-transport limitations.^36–39^ In addition, graphene supports have been shown to alter intrinsic catalytic activity.^30,40^ Alternative configurations, such as silicon nitride (SiN_*x*_) window cells, have been used to eliminate the liquid–vacuum interface while maintaining X-ray transparency. Although SiN_*x*_ windows are capable of maintaining a catalyst–electrolyte interface, they are impermeable to electrons, preventing photoelectrons from escaping to the detector and thus precluding XPS measurements. Consequently, such windowed systems are restricted to X-ray absorption studies and are unsuitable for in situ and operando XPS of electrochemical interfaces. These limitations underscore the need for a window-free, electronically conductive WEA that enables in situ soft X-ray investigations of functional electrocatalysts under realistic reaction conditions.^41–47^

To overcome these limitations, we developed a mesoporous carbon–membrane working electrode assembly (WEA) designed specifically for in situ soft X-ray spectroscopy. The WEA integrates a Nafion proton-conducting membrane with a mesoporous conductive carbon layer that provides both electronic and ionic percolation without the need for any encapsulating window. A thin catalyst layer is deposited directly on the carbon surface, which remains exposed to a controlled water vapor environment during measurement. This configuration allows direct X-ray and electron access to the active surface while maintaining electrochemical hydration and stability. While the current WEA operates at near-ambient pressure and temperature and does not reproduce industrial current densities or gas management, it captures the essential architecture and ionomer/membrane transport characteristics of membrane-based electrolyzers.

Here, we describe the design, optimization, and validation of this mesoporous carbon–membrane WEA for combined electrochemical and soft X-ray studies. We demonstrate that the assembly maintains the necessary charge and mass transport pathways for stable oxygen evolution activity, while simultaneously enabling high-quality Ir 4f and O 1s XPS and NEXAFS spectra under near-ambient conditions. This development bridges the gap between traditional electrochemical cells and vacuum-based spectroscopy, providing a robust and reproducible platform for in situ studies of functional electrocatalysts.

## Experimental Section

Key experimental procedures specific to this study are summarized below. Additional methodological details, including extended experimental protocols, general characterization details, calibration data, fitting (Tables S2–S7), and derivations are provided in the Supporting Information (SI).

### Working Electrode Assembly (WEA) Fabrication

#### Carbon Ink Preparation

Carbon ink was prepared in 100 mg batches using Ketjenblack EC-300J and 22.66 wt % Nafion in a 1:1 carbon:Nafion solids ratio. The mixture was diluted with Milli-Q water to 53 wt % total solids and supplemented with five 3 mm YT2 beads. The ink was speed mixed at 3000 rpm for 5 min, rested for 2 min, and this cycle was repeated twice. The particle size distribution (PSD) was then measured using a Mastersizer 3000 laser diffractometer equipped with a Hydro SV dispersion unit (Malvern Panalytical, U.K.) and controlled by Mastersizer Xplorer software. Measurements were performed using a 22 wt % 1-propanol–water solvent mixture, maintaining laser obscuration between 5 and 10%.

If the PSD exceeded 1 μm, additional 5 min milling steps were applied. The ink was subsequently diluted to 5.3 wt % total solids with 22 wt % 1-propanol, sonicated for 5 min at 50 W (S220, Covaris), and the PSD re-evaluated. If particle sizes remained above 1 μm, the ink was sonicated for 5–10 min at 100–150 W. The final carbon ink concentration was adjusted to 2 mg_carbon_ mL^–1^ using Milli-Q water.

#### Iridium Oxide Ink Preparation

Iridium oxide (IrO_*x*_, Alfa Aesar Premion, 99.99% (metals basis), batch 39689) ink was typically prepared in 100 mg_IrOx_ batches. The IrO_*x*_ powder was first diluted to 61 wt % solids using Milli-Q water and homogenized by speed mixing at 3000 rpm for 30 s. A 22.66 wt % Nafion solution in 1-propanol was then added to achieve a Nafion:IrO_*x*_ solids ratio of 1:10. An additional volume of 1-propanol equal to the initial volume of water was introduced, followed by another 30 s of mixing at 3000 rpm. Five 3 mm YT2 zirconia beads were then added, and the mixture was speed mixed for 5 min at 3000 rpm, rested for 2 min, and this cycle was repeated twice more.

The PSD of the resulting ink was measured using a Mastersizer 3000 laser diffraction particle size analyzer (Malvern Panalytical Ltd., U.K.). If 90% of the volume density exceeded 10 μm, additional 5 min mixing cycles were performed until the PSD was reduced below 1 μm. The ink was then diluted to 5.3 wt % total solids (IrO_*x*_ + Nafion) using 22 wt % 1-propanol and sonicated (S220, Covaris, U.K.) for 5 min at 50 W. The final IrO_*x*_ ink concentration was adjusted to 2 mg_IrOx_ mL^–1^ using Milli-Q water.

#### Spray Coating and Hot Pressing

Immediately before deposition, both inks were sonicated for an additional 30 s to re-disperse the solids. The carbon layer was deposited first, spraying the predetermined ink volume corresponding to the target loading. Direct weighing was avoided due to hydration-dependent mass fluctuations of Nafion with temperature and ambient humidity. The carbon and IrO_*x*_ inks were sequentially spray-coated onto pretreated Nafion 115 membranes. The membrane was secured on a heated vacuum table using a mask to ensure flatness during deposition. The substrate temperature was maintained at 120 °C. A nitrogen carrier gas pressure of 2 bar was applied, with an airbrush–substrate distance of approximately 15 cm and a rastering speed of 2.5 cm s^–1^ to achieve a target coverage of 0.2 or 1 mg cm^–2^ IrO_*x*_ which is confirmed using X-ray fluorescence (XRF) analysis.The completed electrode assemblies were subsequently hot-pressed to ensure adhesion and uniform layer consolidation.

### Electrochemical Characterization

Electrochemical measurements were performed using the B07 spectro-electrochemical cell^5^ in combination with either an Ivium CompactStat potentiostat (Ivium, The Netherlands) equipped with a current booster, or an Autolab PGSTAT30N (Metrohm AG, Switzerland).

Working electrodes were prepared by punching 12- or 10 mm diameter samples from the spray-coated working electrode assemblies (WEAs). The iridium loading of each sample was quantified by X-ray fluorescence (XRF) prior to electrochemical testing to enable current normalization by iridium mass. All measurements were conducted using 0.1 M H_2_SO_4_ as the electrolyte.

High-surface-area Pt was used as the counter electrode, and its preparation is described in the Supporting Information (SI). Different reference electrodes were employed depending on the experimental configuration, as the electrochemical cell was operated either offline (bench setup) or online (mounted on the B07 beamline). For offline electrochemical measurements, a Mini-HydroFlex hydrogen reference electrode (Gaskatel, Germany) was used. For beamline measurements, an Ag/AgCl reference electrode (eDAQ, Australia) was employed, and all potentials were calibrated against the Mini-HydroFlex reference prior to data acquisition.

Cyclic voltammetry (CV) measurements were conducted at a scan rate of 20 mV s^–1^, 4 mV step size, and a 10 mA current range. Electrochemical impedance spectroscopy (EIS) was carried out offline using the Autolab potentiostat in potentiostatic mode with a logarithmic frequency spacing of 13 points per decade over the range 100 kHz to 0.1 Hz. The internal resistance of the cell was determined by fitting the Nyquist plot using semicircular equivalent circuit elements. For internal resistance compensation, if an impedance measurement was not on the sample in the cell made during that series of measurements, an assumed internal resistance value was made using the internal resistance values shown in Figure 3b.

Electrolyte circulation through the spectro-electrochemical cell was achieved using either a Microlab 500B syringe pump (Hamilton, Nevada, USA) or a Spetec Perimax 16 peristaltic pump (Spetec, Germany), depending on the experimental period. For initial experiments—IrO_*x*_ pressure-dependence testing—a 2.5 mL glass syringe (Hamilton) was operated via the Microlab 500B pump. The programmed pumping sequence comprised a 4 s syringe fill followed by a 250 s dispense, corresponding to a net flow rate of 0.01 mL s^–1^. Subsequent experiments (in situ experiments) employed the Spetec Perimax 16 peristaltic pump with 0.254 mm i.d. PVC tubing (part number 38-0015, Spetec). Under standard operating conditions, this setup provided a steady flow rate of 0.03 mL s^–1^, ensuring stable electrolyte replenishment during prolonged in situ XPS and NEXAFS measurements. For all measurements, the electrolyte reservoir contained 100 mL of 0.1 M H_2_SO_4_, which was recirculated continuously throughout the experiment. The same reservoir was used for the entire duration of each measurement series.

### In Situ Experiments

Measurements with electrolyte flow were conducted on beamline B07-C (Diamond Light Source, U.K.) using the TCUP configuration,^5,48^ whereas measurements without electrolyte flow employed the TPOT configuration. Water vapor pressure was precisely controlled via the custom-built water doser, operated in conjunction with a PID-controlled butterfly valve. Potential-hold measurements were performed at 8 mbar water vapor pressure under continuous electrolyte flow.

Pressure-change experiments were carried out during beamtime proposals SI32763-1 and SI36143-1 using an Ivium CompactStat potentiostat (Ivium, The Netherlands). Potential-control experiments were performed during proposals SI34260-1 and SI39495-1 using an Autolab PGSTAT30N potentiostat (Metrohm AG, Switzerland).

Potentials were calibrated versus the reversible hydrogen electrode (RHE) using freshly calibrated reference electrodes immediately prior to each in situ measurement sequence. Oxygen evolution reaction (OER) potential holds were applied at the current density equivalent to approximately 25 g_Ir_^–1^, with the current calculated based on the mass of iridium within the electrochemically active area (defined by the 4.5 mm diameter X-ring sealing the electrolyte cavity). The catalyst mass in this region was determined by X-ray fluorescence (XRF) analysis of the corresponding sample.

### Water Dosing and Pressure Control

The water dosing system (Figure S13) provided a controlled and reproducible supply of water vapor to the near-ambient pressure (NAP) chamber. Liquid water was delivered via a peristaltic pump through a silicone tube terminating in a fine syringe needle, which dispensed water onto a heated silica powder bed. The silica was contained between two stainless-steel meshes, around which a resistive heating wire was coiled. A K-type thermocouple was spot-welded directly to the mesh for temperature monitoring. The mesh temperature was regulated by a Eurotherm PID temperature controller interfaced with a programmable power supply.

A pneumatically controlled valve allowed water vapor generated in the heated silica bed to enter the experimental chamber, while the overall chamber pressure was stabilized by a PID-controlled butterfly valve connected to the vacuum pumping system. The setup enabled stable operation at desired water vapor pressures, typically up to 8 mbar, under continuous electrolyte flow.

### Water Coverage

The thickness of the water overlayer on IrO_*x*_ was determined by fitting the O 1s XPS spectra using the following relationship:

$$A=B\times \frac{e^{-t/\lambda }}{1-e^{-t/\lambda }}$$

where *A* = *I*_IrO_*x*__/*I*_H_2_O_ and *B* = *I*_IrO_*x*__^0^/*I*_H_2_O_^0^. Here, λ_IrO_*x*__ denotes the inelastic mean free path (IMFP) of IrO_*x*_ photoelectrons traveling through the liquid H_2_O overlayer. Solving this expression for ***t*** yields the water-layer thickness.

The peak-area uncertainty of the O 1s XPS measurements is ±0.1, and this value was propagated through the equation to obtain the uncertainty in the calculated water-layer thickness. These propagated errors are shown as the error bars in Figure 5a. A full derivation of the equation is provided in the Supporting Information.

## Results and Discussion

For a geometry in which the electrocatalyst is the outermost layer, enabling the detection of emitted photoelectrons without attenuation by overlayers, both electrochemical and spectroscopic constraints must be satisfied. The WEA must (i) provide a continuous electronic and protonic pathway with minimal ohmic losses, (ii) ensure efficient transport of reactants and products, and (iii) remain in direct or near line-of-sight to the incident X-ray beam and the electron analyzer to maximize signal intensity. Failure to satisfy these requirements would lead to electrochemical and spectroscopic artifacts, rendering the measurements unrepresentative of true reaction conditions.

To meet these requirements, a three-layer WEA architecture (Figure 1) was designed, prepared, and optimized using our newly developed spectro-electrochemical flow cell.^5^ Figure 2 shows a schematic of the in situ setup used for combined XPS and NEXAFS measurements at the B07C beamline of Diamond Light Source.^48^ The flow cell setup incorporates a working electrode (WE; Figure 1) with a layered structure comprising a catalyst + ionomer (1.5 μm), a carbon + ionomer (3 μm), and a proton-exchange membrane (127 μm). Electrolyte (0.1 M H_2_SO_4_) is circulated via a peristaltic pump, while a reference electrode (RE) and counter electrode (CE) enable electrochemical control through a potentiostat. The cell is mounted on the B07C Tea-Cup manipulator equipped with XYZ stepper motors for precise alignment. A water doser and differential pumping at both the X-ray beamline entrance and the analyzer cone enable NAP-XPS and NEXAFS studies of electrocatalytic interfaces.

**Figure 1:**
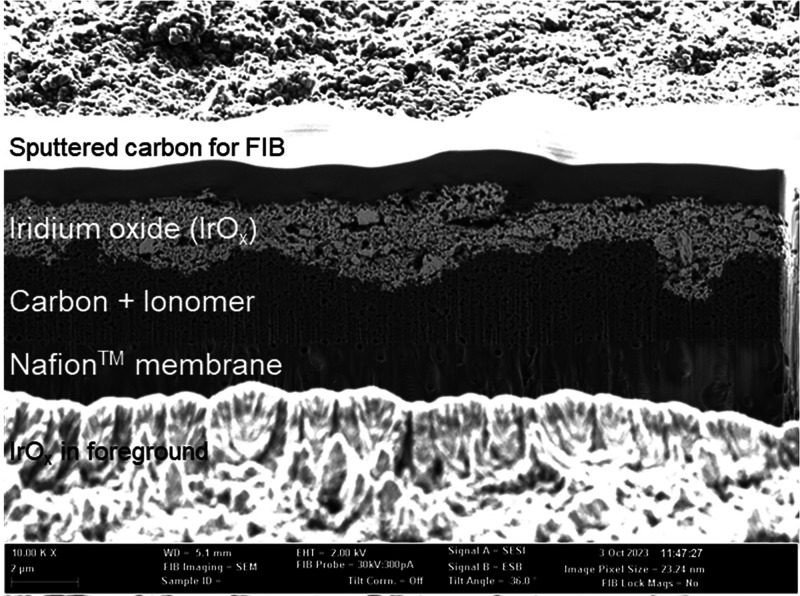
FIB SEM cross section micrograph of a WEA with amorphous iridium oxide.

**Figure 2:**
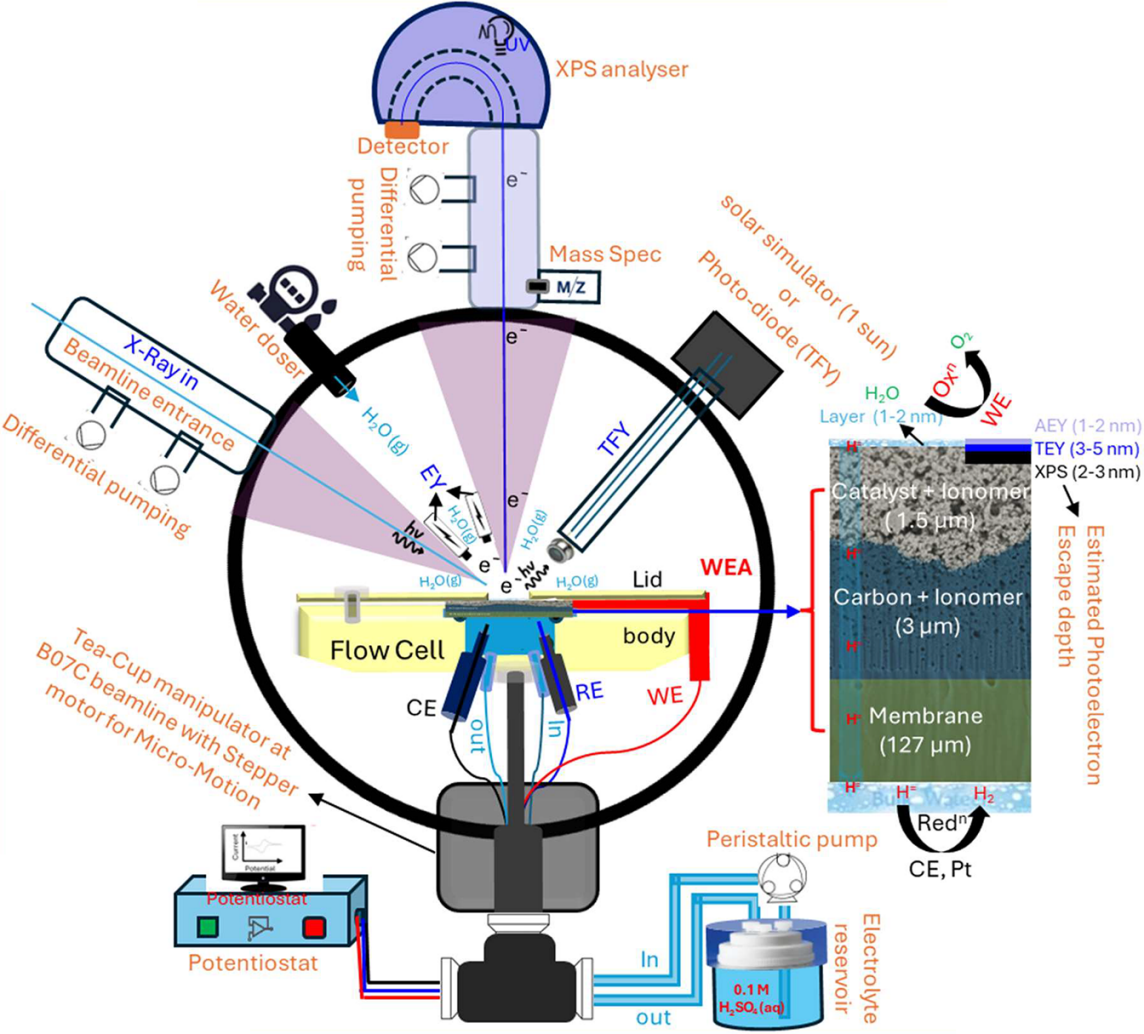
Shows a schematic of the in situ spectro-electrochemical flow cell set up used for combined surface sensitive XPS and NEXAFS measurements under experimental conditions.

XPS and Auger electron yield (AEY) signals were acquired using the hemispherical electron energy analyzer, with AEY obtained by monitoring the Auger electron intensity as a function of incident photon energy. Electron yield (EY) was measured via drain current through either the analyzer cone and/or beamline nozzle. Gas composition in the endstation were monitored using an inline mass spectrometer connected to the analyzer prelens chamber. The B07C beamline additionally supports optical excitation sources, including UV, LED, and solar simulation. Finally, total fluorescence yield (TFY) measurement can be performed using a photodiode, which is shielded to block electrons. Although these capabilities were not employed in the present study, they are fully compatible with the spectro-electrochemical flow cell design and enable future operando photoelectrochemical measurements using the same set up.

The inset in Figure 2 highlights the layered WEA structure together with estimated photoelectron escape depths for Auger electron yield (AEY, 1–2 nm)^49,50^ and total electron yield (TEY, 3–5 nm),^49,50^ underscoring the surface sensitivity of both the XPS and NEXAFS measurements. In the present catalyst-coated membrane configuration, the liquid electrolyte is located on the opposite side of the membrane, with no direct liquid contact to the catalyst layer. This constraint motivates the design and development of the WEA architecture employed in this work. The layered WEA was optimized (discussed in the sections below) to position the catalyst surface on the vacuum-facing side for direct access by the incident X-ray beam and the XPS analyzer, while maintaining electrochemical activity through controlled hydration. Hydration of the vacuum-facing catalyst surface is enabled by the water-permeable membrane, the porous carbon/ionomer and IrO_*x*_/ionomer networks, and external dosing of water vapor in the beamline endstation. The incorporated ionomer plays a crucial role by providing proton-conducting pathways throughout the catalyst layer, thereby enabling electrochemical activity at the vacuum-facing interface despite the absence of direct electrolyte contact. Under these conditions, the detected XPS and NEXAFS signals originate from the electrochemically active hydrated-IrO_*x*_ surface, while the bulk of the ∼1.5 μm-thick catalyst layer remains inaccessible to photoelectrons.

Overall, the WEA design consists of (1) an ion-exchange membrane as mechanical and ionic support, (2) a mesoporous carbon-based conductive layer, and (3) an electrocatalyst layer spray-deposited on top. The ion-exchange membrane (Nafion 115) provides mechanical stability against a 1 bar differential pressure between the cell cavity and endstation, while ensuring high proton conductivity and water permeability with leak control, essential for maintaining hydration at the solid–liquid interface. This configuration closely resembles a half catalyst-coated membrane (half-CCM) used in polymer electrolyte membrane (PEM) electrolyzers and can be adapted to anion-exchange membranes for alkaline or CO_2_ reduction studies.

Two types of conductive underlayers were evaluated: sputtered Au and carbon black. Although sputtered Au films (>20 nm) provided low sheet resistance (∼15 Ω sq^–1^), they suffered from cracking and electrocatalyst delamination upon hydration and handling, ruling them out for practical use. In contrast, mesoporous carbon black layers (2–50 nm pore size) adhered well to the membrane and maintained electrical contact despite membrane swelling. A loading of 320 μg cm^–2^ produced the optimum sheet resistance of ∼160 Ω sq^–1^, reduced by ≈30% after hot pressing (Figure 3a). The flexibility of the carbon network accommodated mechanical expansion and contraction while maintaining conductivity. To balance electron and proton transport, a 1:1 carbon:ionomer weight ratio was used for the conductive layer.

**Figure 3:**
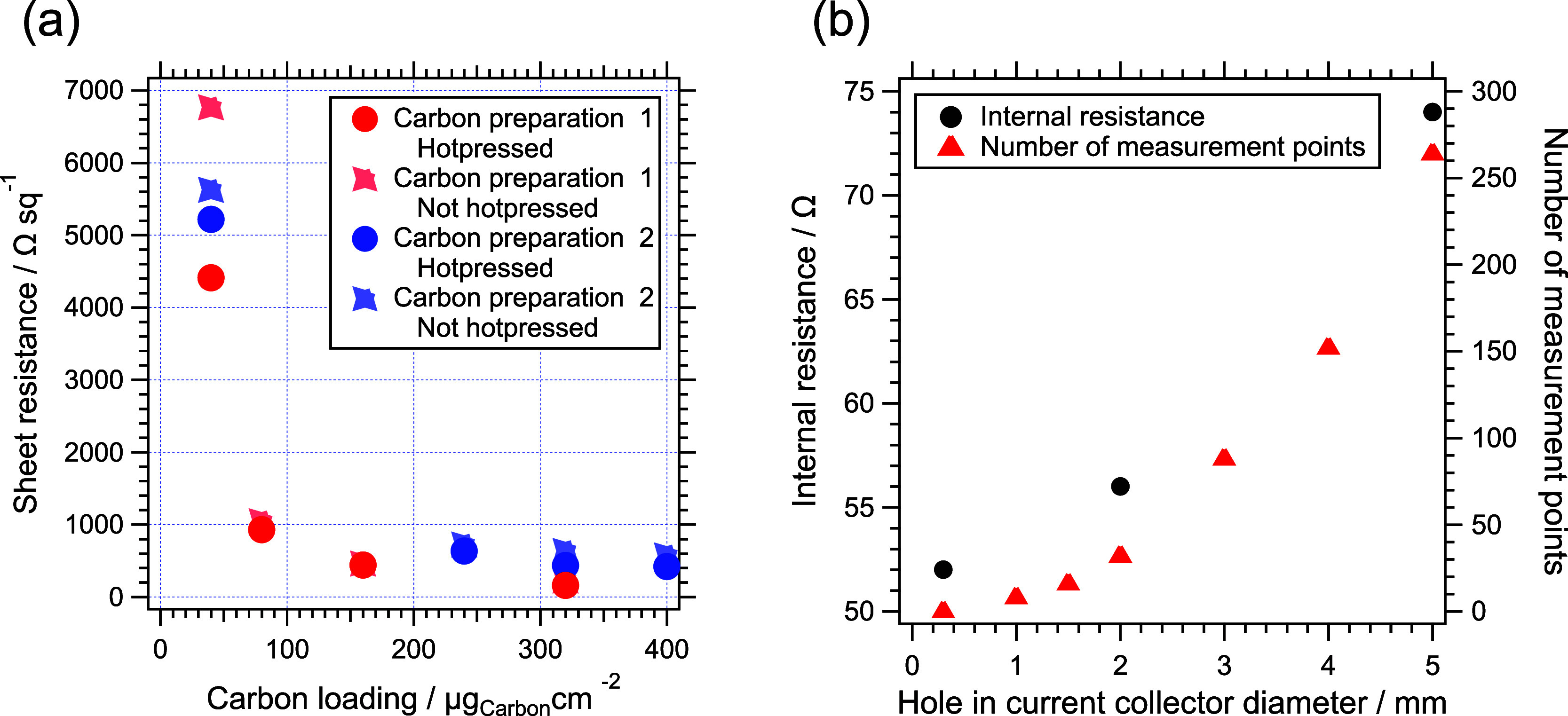
(a) Plot of sheet resistance measured using 4-point probe conductivity against carbon thickness. Carbon preparation series 1 and 2 displayed before and after hot pressing. A version of this plot with a log scaled *y*-axis is shown in Figure S7. (b) Dual axis chart comparing (left) internal resistance values and (right) Number of measurement points, as a function of the diameter hole in the current collector. Internal resistance values from the X-intercept of Nyquist impedance spectra at open circuit voltage (0.466 V vs RHE) of iridium oxide coated WEA.

The electrocatalyst layer (amorphous or crystalline IrO_*x*_) was then spray-coated with 10 wt % Nafion ionomer (1:9 electrocatalyst:ionomer). The 10 wt % ionomer fraction minimizes Nafion contributions to the O 1s XPS region, avoiding overlap with the IrO_*x*_ oxygen signal (Figure S1). This approach also mitigates beam-induced degradation of Nafion, which is evident from F 1s signal loss and reduction in CF_2_ under irradiation (Figure S2 and S3, Table S8). Under in situ water vapor and electrolyte conditions, the Nafion spectral contribution becomes negligible (Figure S4 and Table S9), confirming that the measured spectra predominantly originate from the catalyst layer. Homogeneous layer deposition is critical for uniform electrochemical potential and representative spectroscopy. Gaps in the conductive or catalyst layers lead to increased internal resistance or unwanted signals from underlying carbon or ionomer. Early attempts at drop-casting and spin-coating produced inhomogeneous coatings due to membrane warping and solvent meniscus formation, or significant catalyst wastage, respectively. Consequently, spray-coating was adopted as the most effective technique, enabling rapid solvent evaporation and uniform coverage. X-ray fluorescence (XRF) mapping confirmed slightly higher coverage at the sample centre, consistent with the spray cone profile (Figure S5). Particle size distribution (PSD) control proved critical: inks were processed to achieve D90 (the particle diameter below which 90% of particles fall) < 10 μm, ensuring smooth, homogeneous films (Figure S6).

The influence of the ring-type current collector hole size (Figure S8) on internal resistance was systematically investigated (Figure 3b). A larger opening increases electron path length within the carbon layer, thereby raising resistance. Reducing the aperture diameter from 5 to 2 mm (Figure S8b) lowered the internal resistance from 74 to 56 Ω, while further reduction to 0.3 mm yielded only a modest improvement (52 Ω) but significantly limited the exposed catalyst area for analysis. Conversely, eliminating the hole entirely caused resistance to rise to 65 Ω, attributed to mechanical wrinkling of the membrane constrained by the unperforated current collector.^46–48^ Lateral potential variations within the illuminated region were also evaluated. In situ valence-band XPS measurements at multiple lateral positions (centre and 0.2–0.4 mm offsets) showed no measurable shift in the valence-band edge (Figure S9). The continuous, laterally conductive carbon underlayer efficiently distributes electrons from the catalyst surface to the ring-type current collector (Figure S8), ensuring uniform potential across the probed area. Theoretical estimates based on sheet resistance (∼11 mV at 10 mA cm^–2^ (eq S3);^51^ provide a conservative upper bound but overestimate the actual lateral potential drop due to the measurement positions near the edge of current collector hole. This confirms that the WEA design maintains adequately uniform electrochemical conditions across the illuminated catalyst area, ensuring reliable XPS and NEXAFS measurements. The 2 mm configuration thus provided the best balance between electrical performance and spectroscopic accessibility.

To complement the WEA, a high–electrochemical-surface-area (ECSA) counter electrode was fabricated to ensure reversible charge balance under the constrained geometries of the flow cell. A Pt wire (Figure S10a) or rod (Figure S10b) was coated with nanostructured Pt of single-diamond (Fd3m) symmetry, resulting in a 110-fold increase in accessible surface area, corresponding to an ECSA of 101 cm^2^ and a mass utilization of 4.19 m^2^ g^–1^. The cyclic voltammetry (Figure S10c–e) shows a dramatic enhancement in current response and clear H-adsorption features, confirming the availability of multiple Pt facets with varied Pt–H binding energies (Table S10). The improved counter electrode performance ensures stable operation during OER and eliminates mass-transport limitations in the confined geometry of the in situ cell.

The electrochemical performance of the WEA was validated using cyclic voltammetry (CV) and linear sweep voltammetry (LSV) in 0.1 M H_2_SO_4_ under working conditions on the beamline (Figure 4). The IrO_*x*_-coated WEA exhibited characteristic features of amorphous iridium oxide (Figure 4a):^52–54^

- 0.8 V: Ir^3+^ → Ir^4+^ oxidation (feature I)
- 1.1–1.4 V: further oxidation towards Ir^5+^ alongside deprotonation of hydroxides (feature II),
- >1.4 V: oxygen evolution reaction (OER) (feature III)
- 1.4–1.1 V (reverse sweep): reduction towards Ir^4+^ alongside protonation of oxides (feature IV)
- 0.7 V (reverse sweep): Ir^4+^ → Ir^3+^ reduction (feature V)

**Figure 4:**
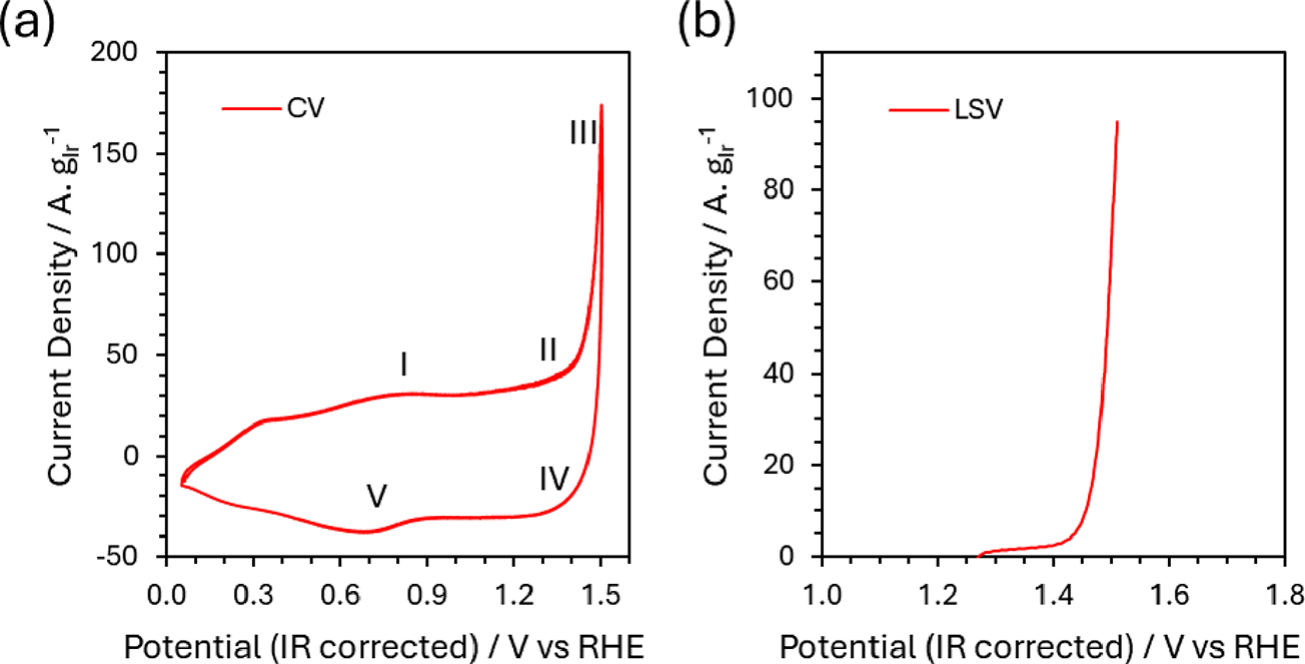
IR corrected (a) cyclic voltammogram recorded from 0.06 to 1.52 V vs. RHE and (b) linear sweep voltammogram of the WEA with amorphous IrO_*x*_ at 1.52 V vs. RHE; the data were recorded on the beamline under in situ working conditions at 8 mbar of water vapor pressure in the end station with 0.1 M H_2_SO_4_ electrolyte flowing in the cell continuously at 2 mL min^–1^. CV and LSV sweep rate 20 and 5 mV s^–1^, respectively; 2 mm diameter current collector hole; internal resistance corrected potential vs. RHE.

Previous in situ XAS studies have suggested that the first and second redox transitions in similar amorphous IrO_*x*_ correspond to the Ir^3+^/Ir^4+^ and Ir^4+^/Ir^5+^ couples, respectively.^41–47^ However, a consensus on the exact oxidation states, particularly under OER conditions, has not yet been reached.^27^ In the OER potential region associated with the third redox transition, in situ XAS and XPS studies have proposed the presence of Ir^4+^,^46^ Ir^4x+^,^47^ or Ir^5+^ species.^10^ ^,^ ^48,49^ More recent studies have further suggested the formation of electrophilic oxygen species (O^1–^) at OER-relevant potentials.^20,21,26^

LSV of the amorphous IrO_*x*_ electrode (Figure 4b) in 0.1 M H_2_SO_4_ electrolyte solution shows a gradual increase in anodic current with applied potential, followed by a pronounced rise corresponding to the onset of the oxygen evolution reaction at 1.47 V vs RHE. The pre-OER region is characterized by a broad anodic current response, attributed to potential-dependent Ir–O redox processes and pseudocapacitive behavior of the amorphous IrO_*x*_ as seen in CV. At higher potential, the rapid increase in current reflects sustained OER activity under steady-state in situ conditions using in situ cell. The stability of the electrochemical response under OER-relevant conditions is further illustrated by Figure S11a, which shows stable current density and potential as a function of time during potentiostatic operation.

As shown in Nyquist plots (Figure S11b) obtained from electrochemical impedance spectroscopy (EIS), the charge-transfer resistance increases from approximately 75 Ω for the carbon-only electrode to approximately 100 Ω for the IrO_*x*_ + carbon electrode at 0.8 V. At this potential, the dominant faradaic processes are not associated with the oxygen evolution reaction, and the increased charge-transfer resistance therefore reflects changes in interfacial reaction kinetics rather than electronic conductivity. IrO_*x*_ is not expected to exhibit fast kinetics for these low-potential processes, which likely explains the observed increase in charge-transfer resistance. Importantly, the high-frequency resistance remains low, indicating that the IrO_*x*_–carbon WEA maintains good electronic contact and ionic connectivity. Overall, these results validate the electronic, ionic, and geometric suitability of the WEA for in situ NAP-XPS and NEXAFS studies under true reaction conditions.

Stable and reproducible electrolyte management was central to the design of the in situ spectro-electrochemical flow cell. Continuous electrolyte flow was required to maintain continuous flow, help mitigate bubbles around electrodes, deliver reactants, and purge evolved H_2_ bubbles from the counter electrode. Early experiments employed a Hamilton Microlab 500B syringe pump (flow rate ≈ 0.01 mL s^–1^; 4 s fill, 250 s dispense). Although this configuration enabled >24 h unattended operation, periodic oscillations in current density (∼90 s rise followed by sharp drops with a 10–12 min period) indicated mechanical asymmetry in the switch from pull to push in the refill cycle, which induced transient sample motion and spectral noise (Figure S12). Replacing the syringe pump with a Spetec Perimax 16 peristaltic pump and 0.254 mm i.d. PVC tubing (flow rate ≈ 0.03 mL s^–1^) eliminated these oscillations and yielded continuous, pulse-free flow. The peristaltic system is better suited due to its ease of automation and continuous flow, ensuring stable operation with 100 mL of recirculating 0.1 M H_2_SO_4_ solution. Electrolyte was argon-degassed for 1 h prior to use, with the headspace continuously blanketed in Ar to prevent re-oxygenation and bubble formation.

To regulate the vapor phase under near-ambient pressure (NAP) conditions, a custom water-dosing unit was developed (Figure S13). The design comprised a heated alumina powder-bed evaporator (60–80 °C) fed by a controlled 0.01–0.025 mL min^–1^ liquid water stream from the peristaltic pump. The vapor output was coupled to the analysis chamber through a pneumatically actuated valve and PID-controlled butterfly throttle, providing precise pressure control. Typical operation achieved 6–10 mbar H_2_O with ±0.1 mbar stability sustained for >2 h. Stepwise transitions (e.g., 10 → 6 mbar) were reproducible, and extended holds revealed only minor drift from condensation in the exhaust lines, which was reversible by N_2_ purge. Together, the alumina-bed doser, peristaltic electrolyte circulation, and closed-loop pressure control define a reproducible hydration environment for in situ NAP-XPS and NEXAFS.

To satisfy the dual requirements of maintaining sufficient water coverage on the electrocatalyst surface for electrochemical activity while minimizing attenuation of photoelectrons for XPS and NEXAFS measurements, the WEA was systematically optimized as a function of water vapor pressure, and both the spectroscopic and electrochemical responses were systematically investigated as a function of water vapor pressure.

The current response of crystalline iridium oxide as a function of pressure (Figure 5a) indicates that the catalyst is underutilized without externally dosed water vapor in the chamber (beamline endstation). At 1 V vs RHE, where the current is dominated by capacitive charging, a linear increase in capacitance current is observed between 6 and 9 mbar, with a gradient of 0.02 mA cm^–2^ mbar^–1^. The slope decreases slightly above 9 mbar, suggesting near-saturation of the interfacial hydration layer. At 1.6 V, where OER occurs, a pronounced rise in current density of 0.088 mA cm^–2^ is observed between 6 and 8 mbar, followed by a smaller increase of 0.037 mA cm^–2^ from 8 to 10 mbar. These results indicate that catalyst utilization increases significantly between 6 and 8 mbar, but only marginally at higher pressures. Very interestingly, electrolyte flow through the cell also has a substantial effect on the amount of water adsorbed on the electrocatalyst surface, as observed from a comparison of the O 1s XPS spectra measured as a function of water vapor pressure with and without electrolyte flow (Figure S14). At 10 mbar water vapor pressure, approximately 5 nm of water is adsorbed when electrolyte is flowing, compared with <0.5 nm in the absence of flow (Figure 5a and eqs S3–S18). This demonstrates that water permeates through the Nafion membrane and mesoporous carbon-ionomer layer. In combination with externally dosed water vapor, this maintains a nanometer-thin hydration layer at the catalyst surface, balancing water evaporation and re-adsorption under vacuum conditions.

**Figure 5:**
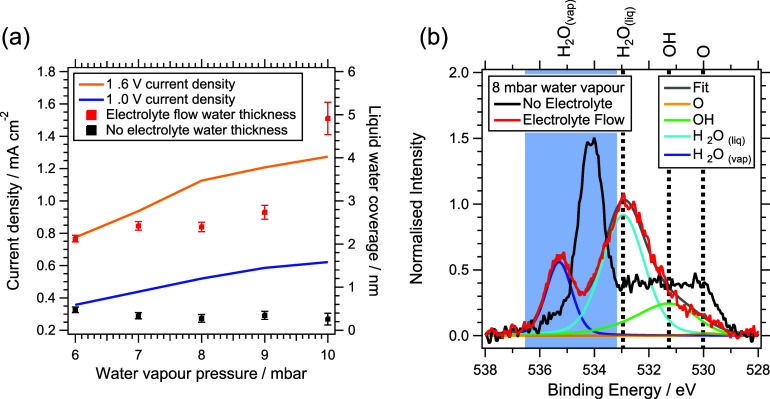
(a) Dual axis chart left axis displays current densities of crystalline IrO_2_ at 1 and 1.6 V during the anodic sweep on the beamline, Right axis liquid water thickness as a function of water vapor pressure (*x*-axis). (b) Comparison of O 1s XPS spectra at 8 mbar water vapor pressure with and without electrolyte flow behind the sample.

While higher water vapor pressures lead to greater catalyst utilization, the resulting increase in water layer thickness and gas-phase density attenuates the XPS signal from the catalyst, as shown in Figure 5b and Figure S14. Under electrolyte flow, the oxide (BE = 529.95 eV) and hydroxide (BE = 531.5 eV) peaks are substantially reduced, whereas the liquid water peak (BE = 532.9 eV) becomes more pronounced, reflecting enhanced interfacial hydration. The water vapor peak in O 1s XPS therefore arises from gas-phase water generated by electrolyte permeation through the Nafion membrane and stabilized by the chamber water vapor, establishing a nanometer-thin hydration layer at the catalyst surface. This layer enables simultaneous electrochemical activity and surface-sensitive XPS/NEXAFS measurements. The IrO_*x*_ layer is not fully immersed in electrolyte; rather, electrolyte flow and chamber hydration together maintain a vapor- or liquid-exposed surface, reflected in the increased intensity of the liquid water peak in O 1s XPS. These observations confirm that the WEA configuration enables probing of the catalyst–electrolyte interface under realistic reaction conditions. To obtain high-quality in situ spectra while maintaining electrochemical activity, an appropriate balance must be achieved between surface hydration and photoelectron attenuation. Based on these coupled electrochemical and spectroscopic considerations, a water vapor pressure of 8 mbar was selected as an optimal operating condition for in situ measurements in this work.

It is worth noting that the electron analyzer cone is biased to attract secondary electrons, modifying the local electrostatic environment near the sample surface. Variations in working distance and sample orientation can therefore influence the apparent position and intensity of the gas-phase peak. The intensity of this peak depends on the the local gas pressure and the fraction of photoelectrons captured by the analyzer; thus, changes in chamber pressure, sample position, and electrode bowing under differential pressure influence the apparent gas-phase contribution. Because the XPS spectra are charge-referenced to the sample rather than the vacuum level, these effects primarily alter the width and position (BE = 533.4–536.5 eV) of the water vapor peak rather than the relative binding energies of surface species (Figures 5b and S14).

Collectively, these results validate the mesoporous carbon–membrane WEA as a robust and optimized platform for in situ soft X-ray spectroscopy: it maintains electrochemical functionality under near-ambient conditions while providing a reproducible, nanometer-scale hydration layer that is spectroscopically transparent.

### In Situ Spectroscopy of the Water–IrO_*x*_ Interface under OER Conditions

To validate the performance of the electrochemical flow cell and demonstrate its capability for in situ soft X-ray spectroscopy under electrochemical control, the water–IrO_*x*_ (amorphous) interface was examined during the oxygen evolution reaction (OER). In situ measurements were conducted at a constant current density of ≈25 A g_Ir_^–1^, corresponding to an approximate potential of 1.47 V vs RHE, ensuring uniform catalytic activity and a stable potential across the working electrode during data acquisition. Reference spectra were collected at 0.5 V vs RHE, a potential below the onset of further potential-induced Ir oxidation and surface deprotonation thresholds.

The Ir 4f XPS spectra (Figure 6a and Table S11) show a clear shift in iridium oxidation state between baseline and OER conditions. At 0.5 V, Ir is present predominantly as Ir^4+^, with a minor Ir^3+^ contribution, with relative contributions of 94.3 and 5.7% and corresponding binding energies of 61.2 and 62.0 eV, respectively. Under OER conditions, these features shift to higher binding energies of 62.0 and 63.0 eV, consistent with the formation of Ir^4+^ and Ir^5+^ species, contributing 53.1 and 46.9% of the total Ir 4f signal, respectively. This evolution is consistent with the anodic Ir-centered redox processes observed in the cyclic voltammetry (Figure 4a), indicating that the WEA preserves genuine electrochemical behavior under X-ray illumination. The emergence of a substantial Ir^5+^ component provides spectroscopic evidence for highly oxidized iridium species that have long been proposed to participate in OER catalysis on IrO_*x*_.^55,56^ Its detection under steady-state conditions reinforces the mechanistic model involving lattice oxygen involvement and validates the cell’s sensitivity for resolving active electronic states at the solid–liquid interface.

**Figure 6:**
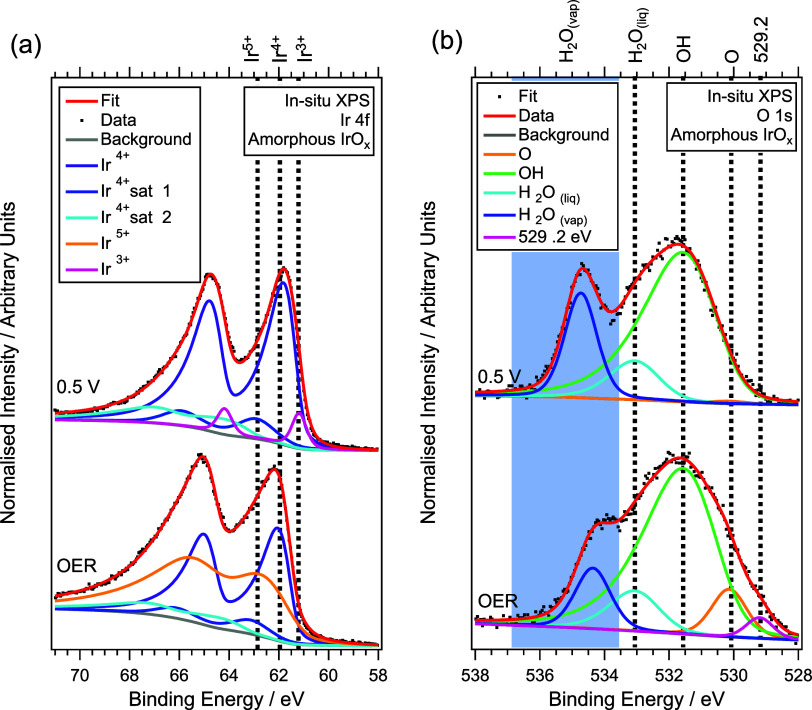
Fitted XPS spectra of amorphous IrO_*x*_ at 0.5 V and under OER conditions. (a) Ir 4f (b) and O 1s.

The O 1s XPS spectra (Figure 6b and Table S12) provides complementary insight into surface deprotonation and oxygen speciation under applied potential. At 0.5 V, the surface is dominated by hydroxyl species (BE ≈ 531.5 eV), accounting for 86.8 % of the total fitted O 1s peak area within the XPS probing depth, with a minor liquid water contribution (BE ≈ 533.0 eV, 12.6%). Under OER applied potential, the hydroxyl signal decreases to 74.2% and liquid water signal to 12.4%, while the oxide component increases to 8.5 %, consistent with progressive −OH deprotonation and the formation of Ir–O–Ir bridge (μ_2_–O, BE ≈ 530.0 eV) species. It is noted that these assignments reflect the near-surface chemical environment sampled by XPS under hydrated conditions. These changes are in agreement with the iridium oxidation state change shown in Figure 6a and the fact that the catalyst has gone through several redox peaks in the CV data. In addition, there is also a low-binding-energy feature at 529.2 eV emerges under OER conditions, attributed to an electron-deficient species with O 2p hole character (oxyl like μ_1_–O^δ‑^) associated with the electrophilic OER pathway.^28,54^ Because the measurements in this work are performed under steady-state conditions, this feature is interpreted as a potential-stabilized oxygen species.

O K-edge NEXAFS spectra (Figure 7a and Table S13) further resolve the coordination environment of active oxygen species under applied potential. The higher-energy region of the O K-edge is dominated by water vapor, which obscures contributions from oxide, hydroxide, and liquid water species; therefore, our analysis focuses on the pre-edge region, while the full spectra are provided in Figure S15. At OER potentials, new pre-edge features appear at 528.5, 529.0, and 529.9 eV, corresponding to μ_1_–O (terminal Ir–O), μ_2_–O (bridging Ir–O–Ir), and μ_3_–O/μ_2_–OH (bulk lattice oxygen) species, respectively.^28,54,57^ The difference between the total electron yield (TEY) and Auger electron yield (AEY) detection modes further clarifies spatial localization. AEY detection (Figure 7b and Table S13) samples inelastically scattered Auger electrons with a shorter escape depth than TEY detection, shows a 11 % higher μ_2_–O contribution than TEY. More specifically, at 0.5 V, the μ_2_–O/μ_3_–O ratio increases from 0.29 in TEY to 0.35 in AEY, while under OER conditions this ratio increases more markedly from 0.94 (TEY) to 1.45 (AEY). The enhanced μ_2_–O/μ_3_–O ratio in AEY, particularly under OER conditions, indicates an increased population of deprotonated bridging lattice oxygen species in the near-surface region relative to bulk-like oxygen environments. The μ_1_–O and μ_2_–O intensities increase with applied potential in both TEY and AEY, with a more pronounced increase observed in AEY, reflecting a greater near-surface oxygen activation. The μ_1_–O feature is therefore associated with a potential-dependent oxygen configuration that becomes increasingly populated under anodic applied potential, while μ_2_–O is consistent with a more robust bridging lattice oxygen environment that remains present under OER-relevant steady-state conditions. The simultaneous presence and systematic evolution of both features with potential indicate a coupled response of surface-exposed μ_1_–O and near-surface bridging μ_2_–O during OER.

**Figure 7:**
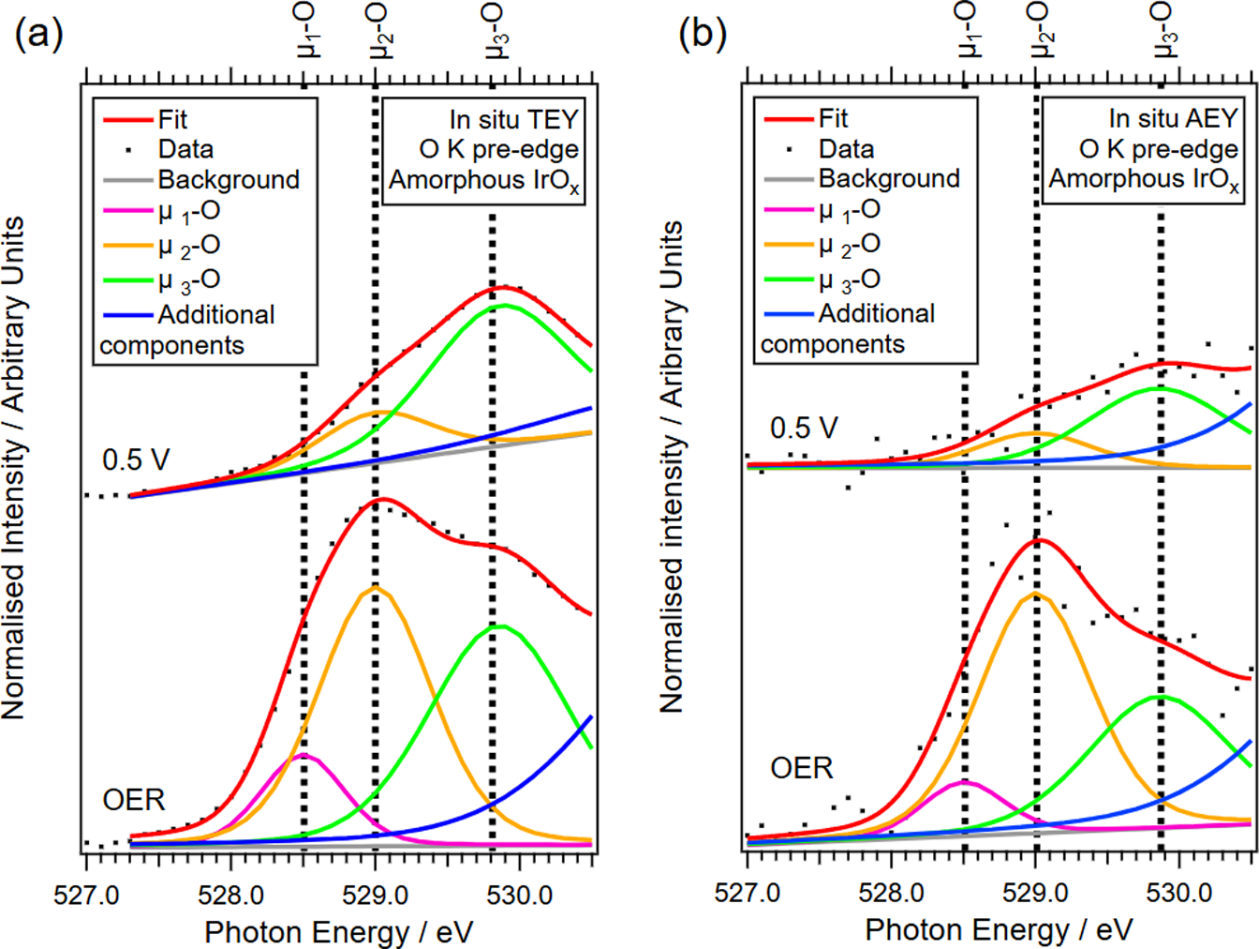
Fitted pre-edge region of O K-edge NEXAFS spectra at 0.5 V and OER (a) TEY detection mode, (b) AEY detection mode.

Together, these results demonstrate a clear correlation between iridium oxidation states and potential-dependent oxygen speciation on amorphous IrO_*x*_ under OER. The concurrent rise of Ir^5+^ states (Ir 4f XPS) and the growth of μ_2_–O species are consistent with a mechanistic framework involving oxidation of Ir^4+^ centers to higher valence states, indicating subsurface lattice oxygen activation. In contrast, the more potential-sensitive evolution of μ_1_–O species also suggests an association with localized surface oxygen configurations that become increasingly populated under anodic applied potential. The simultaneous presence and systematic evolution of both μ_1_–O and μ_2_–O species within the same potential window indicate a coupled response of surface and lattice oxygen environments during OER, reflecting a redistribution of oxygen character under steady-state electrocatalytic conditions. A detailed mechanistic analysis of the role of individual oxygen species is beyond the scope of the present study. The observations reported here are therefore limited to potential-dependent changes in iridium and oxygen speciation under electrochemical applied potential. Follow-up work will systematically investigate the evolution of μ_1_–O and μ_2_–O species in relation to Ir oxidation states as a function of potential, crystallinity, and electrochemical aging, to refine the mechanistic understanding and assess their role in activity and stability.

## Conclusions

A mesoporous carbon–membrane working electrode assembly (WEA) has been developed and validated for in situ soft X-ray spectroscopy of electrochemical interfaces. The architecture combines a Nafion proton-conducting membrane with a mesoporous carbon–ionomer network and a window-free IrO_*x*_ catalyst layer, enabling concurrent electron, proton, and mass transport while maintaining direct X-ray access. Integration with a peristaltic electrolyte delivery system, high-surface-area Pt counter electrode, and stabilized micro-reference provides robust electrochemical performance. A closed-loop, heated alumina-bed doser maintains stable water vapor pressure (6–10 mbar) required for NAP-XPS and TEY-NEXAFS measurements. At an optimal 8 mbar water vapor pressure, the WEA sustains a nanometer-thin hydration layer sufficient for the oxygen evolution reaction (OER) while preserving high-quality XPS and NEXAFS signals. In situ spectra reveal potential-dependent oxidation of Ir^3+^/Ir^4+^ to Ir^4+^/Ir^5+^ and the evolution of potential-stabilized μ_1_–O and μ_2_–O oxygen ligand species. This simple, window-free design provides a robust, vacuum-compatible platform for qualitative and quantitative in situ spectroscopy of electrocatalytic interfaces. The approach can be readily adapted to other membranes and reaction environments, opening new opportunities for mechanistic studies of electrochemical energy-conversion materials.

## Supplementary Material



## Notes

Additional experimental details, electrochemical data, beamline configurations, and raw XPS/NEXAFS spectra (PDF)
