# Supplementary material for: A Catalyst-Coated Mesoporous Carbon–Membrane Electrode Assembly for In Situ Soft X‑ray XPS and NEXAFS Studies of Electrocatalytic Interfaces
Source: ACS Electrochem. 2026 Mar 17;2(5):1226–37. doi: 10.1021/acselectrochem.5c00554 (PMC13158919; doi:10.1021/acselectrochem.5c00554)
Supplement: Supplementary file 1 [file ec5c00554_si_001.pdf]

## Supporting Information

# A Catalyst-Coated Mesoporous Carbon–Membrane Electrode Assembly for In situ Soft X-ray XPS and NEXAFS Studies of Electrocatalytic Interfaces

James J. C. Counter,†‡ Santosh Kumar,†* Christopher M. Zalitis,§ Mark Clapp,§ Alexander I. Large,† David C. Grinter,† Matthijs A. van Spronsen,† Pilar Ferrer,† Burcu Karagoz,† Tugce Eralp Erden,§ Roger A. Bennett,‡ and Georg Held,†*

† Diamond Light Source, Harwell Science and Innovation Campus, Didcot, Oxfordshire OX11 0DE, United Kingdom
‡ Department of Chemistry, University of Reading, Reading RG6 6DX, United Kingdom
§ Johnson Matthey Technology Centre, Blounts Court Road, Sonning Common, Reading RG4 9NH, United Kingdom

* Corresponding Authors: santosh.kumar@diamond.ac.uk; georg.held@diamond.ac.uk

# Contents

Additional experimental details ..... S3

Pt Counter Electrode Preparation ..... S3

Reference Electrode Calibration ..... S4

General Characterization ..... S4

Sheet Conductivity Measurements ..... S4

Scanning Electron Microscopy (SEM) and Energy-Dispersive X-ray Spectroscopy (EDS) ..... S4

X-ray Fluorescence Spectroscopy (XRF) ..... S4

XPS and NEXAFS Measurements ..... S5

XPS Fitting ..... S6

NEXAFS Pre-edge Fitting ..... S10

Supplementary Figures ..... S12

Water doser ..... S23

Water coverage derivations ..... S24

In situ spectroscopy results ..... S27

References ..... S28

# Additional experimental details

## Pt Counter Electrode Preparation

High-surface-area Pt counter electrodes were fabricated following the method of Akbar et al. (2013).[1] A PalmSens EmStat3 Blue potentiostat (PalmSens, The Netherlands) was used with a Pt mesh counter electrode and a saturated Ag/AgCl reference electrode. Electrode behavior before and after Pt deposition was assessed by cyclic voltammetry (CV) in 0.5 M $H_2SO_4$ at 10 mV $s^{-1}$. Following initial CV characterization, the electrode was rinsed and dip-coated in a phytantriol–ethanol (1:2 w/w) mixture and left for 2 h to evaporate ethanol.

The electrode was then immersed for 10 min in 8 wt % aqueous hexa-chloroplatinic acid (HCPA) (Merck, p.a. grade, diluted with Milli-Q water) at room temperature, followed by electrodeposition at −0.2 V vs Ag/AgCl. During this step, the residual phytantriol self-assembled into an inverse bicontinuous cubic phase, acting as a nanostructured template for Pt nanowire growth.[2] After deposition, electrodes were rinsed three times with ultrapure water, three times with 1:1 ethanol–water and then soaked overnight in 1:1 ethanol–water to remove the phytantriol. Post-treatment CVs confirmed successful nanowire formation.

The **electrochemical surface area (ECSA)** was determined from the hydrogen adsorption region of the CV according to Doña Rodríguez et al. (2000),[3] using the Pt surface charge density reported by Biegler et al. (1971).[4]

The mass of deposited Pt was calculated via eqn S1:

$$m_{Pt} \frac{\int (I\,dt) \times A_r(Pt)}{4 \times e \times N_A} \qquad \text{S1}$$

Where $m_{Pt}$ is the mass of Pt electrodeposited (g), I is the current from the deposition transient (A),$A_r$(Pt) is the molar mass of Pt (g mol$^{-1}$), e is the elementary charge (C),[5] $N_A$ is the Avogadro constant.

The surface area per gram is calculated by eqn S2:

$$C_{Pt} = \frac{S_{Final} - S_{Initial}}{m_{Pt}} \qquad \text{S2}$$

Where $C_{Pt}$ is the electrochemical surface area of Pt per gram (m$^2$ g$^{-1}$), $S_{Final}$ is the electrochemical surface area of Pt after deposition (m$^2$), $S_{Initial}$ is the electrochemical surface area of Pt before deposition (m$^2$).

## Reference Electrode Calibration

All reference electrodes were calibrated against a Mini-HydroFlex hydrogen reference electrode (Gaskatel, Germany), which was stored in 0.1 M $H_2SO_4$ and refreshed annually with a new hydrogen cell. To prevent contamination, this electrode was used exclusively for calibration.

Redox.me reference electrodes were stored in saturated AgCl solution, while eDAQ reference electrodes were stored in 0.1 M $H_2SO_4$. Before and after each use, all electrodes were rinsed three times with deionized water.

For calibration, electrolyte from the cell reservoir was pumped through the reference chamber, and electrodes were allowed to equilibrate for 10 min before potential readings were recorded. During in situ experiments, reference electrode potentials were verified at both the start and end of each measurement.

Initial in situ measurements on pristine $IrO_x$ films employed Redox.me reference electrodes, while subsequent measurements on aged samples used eDAQ electrodes following their implementation in later experiments.

## General Characterization

### Sheet Conductivity Measurements

Sheet resistance ($\Omega\ sq^{-1}$) was determined using a Lotersta-GX four-point probe with an LSP head (Nittoseiko Analytech, Japan). The conductive area for each sample was defined by the mask area used during spray coating. Measurements were performed five times per sample, with the probe orientation varied between each measurement. The mean average of these five measurements was reported as the sheet resistance value.

### Scanning Electron Microscopy (SEM) and Energy-Dispersive X-ray Spectroscopy (EDS)

Surface and cross-sectional morphologies were analyzed using a Crossbeam 550 scanning electron microscope (Zeiss, Germany) equipped with both secondary electron and backscattered electron detectors. Cross-sections were prepared in situ within the microscope using focused ion beam (FIB) milling. Prior to milling, a thin carbon protective layer was sputter-deposited onto the surface. Milling was then performed with a focused gallium ion beam.

Energy-dispersive X-ray spectroscopy (EDS) was carried out at selected SEM analysis sites using an X-Max 150 $mm^2$ detector (Oxford Instruments, United Kingdom) operated in conjunction with Aztec data processing software.

### X-ray Fluorescence Spectroscopy (XRF)

X-ray fluorescence spectroscopy was used to determine the precious metal loading on the working electrodes. Measurements were performed using a Fischerscope X-ray XDV-SDD instrument (Helmut Fischer GmbH, Sindelfingen, Germany) with a 0.6 mm collimator and a 15 s acquisition time under automatically optimized anode current conditions. To minimize

background contributions, samples were suspended on a grid above the instrument baseplate, providing a 10 cm air gap below the sample.
Before each series of measurements, background spectra were acquired using membranes coated only with the conductive layer to isolate the contribution from the electrocatalyst. For each electrode, five individual XRF measurements were recorded, and the mean value was used to calculate the deposited metal loading. These data were subsequently employed to normalize current densities and calculate catalyst mass activities.

**XPS and NEXAFS Measurements:** X-ray photoelectron spectroscopy (XPS) and near-edge X-ray absorption fine structure (NEXAFS) experiments were conducted on the B07-C soft X-ray beamline at the Diamond Light Source.[7] Both techniques were performed sequentially for each experimental condition.

A PHOIBOS 150 NAP electron energy analyzer (SPECS GmbH, Germany) was used for all XPS measurements. The electrically isolated cone of the analyzer simultaneously served as the electron collector for total electron yield (TEY) NEXAFS measurements, with a +5 V bias applied to the cone to attract emitted electrons. For XPS, the monochromator was operated in CFF 2 mode using a 600 lines $mm^{-1}$ grating. The analyser is oriented such that its axis is at ∼60° to the incident X-ray beam and ∼30° to the horizontal, with a working distance of ∼0.3–0.45 mm between the analyser cone and the sample surface —parameters that define the collection geometry and influence surface sensitivity. Furthermore, the incident soft X-ray photon flux at the sample was estimated from reference Au 4f measurements under vacuum, considering the analyzer geometry, pass energy, and sampling depth. Based on these measurements, the photon flux is on the order of $10^{10}$–$10^{11}$ photons $s^{-1}$. Under the operando measurement conditions at 8 mbar $H_2O$, electron scattering slightly reduces the detected signal, but the incident flux remains consistent with the VerSoX B07 beamline specifications, providing sufficient intensity for operando XPS and NEXAFS studies.

The number of iterations was adjusted to achieve acceptable signal-to-noise ratios. Binding energy (BE) windows were modified as required to capture the spectral regions of interest and accommodate small shifts caused by sample charging. Typically, charge compensation was achieved by measuring the Fermi edge at each analysis position and photon energy, followed by aligning all spectra relative to the measured Fermi level. For samples lacking a distinct Fermi edge (e.g., carbon layers), the C 1s core-level peak was used for charge calibration at 284.8 eV.

The photon energies (1070 and 1540 eV) were chosen such that the kinetic energy of emitted photoelectrons from the Ir 4f and O 1s regions was approximately 1000 eV, corresponding to an inelastic mean free path (IMFP) of 19.2 Å (calculated via the TPP-2M equation). This yields an information depth of 57.6 Å, probing roughly 140 atomic layers in rutile $IrO_2$.[8,9]

For the O 1s and Ir 4f regions, the kinetic energies and thus information depths were equivalent, enabling direct comparison of oxidation-state-dependent changes. Beamline exit slits of 0.05 mm were employed, providing resolutions of <0.55 eV for Ir 4f and <0.9 eV for O 1s spectra. XPS spectra were Fermi-edge calibrated using the corresponding valence band

(VB) spectra. Where spectra background normalised at the low BE point for the region shown in **Table S1** at the low energy, then Shirley background subtracted between the low and high BE points (with the exception of survey spectra) shown in **Table S1**.

**Table S1**. BE positions for normalisation and background subtraction of XPS spectra.

| Region | Low binding energy / eV | High binding energy / eV |
|---|---|---|
| Ir 4f | 57 | 72 |
| C 1s | 280 | 294 |
| O 1s | 527 | 540 |
| Survey | 45 | |

Data analysis and peak fitting were performed using Igor Pro 9 (Wavemetrics, USA) with the XPST package developed by Dr. Martin Schmid (Philipps University, Marburg).

For O K-edge NEXAFS, exit slits of 0.1 mm were used, yielding a resolution of <0.475 eV. Auger electron yield (AEY) spectra were acquired with a 160 eV pass energy. The photon energy scale was calibrated to a known spectral feature in the O K-edge of the gold mesh used in the beam path. NEXAFS data were normalized by dividing the sample signal by the gold mesh transmission signal to account for beamline transmission fluctuations.

Background intensity was determined at 527 eV, below the O K-edge onset, and subtracted prior to normalization. The spectra were then normalized to 1 with respect to the intensity at 550 eV. Since only $IrO_x$ contributes to the pre-edge region, this procedure effectively compensates for variations in sample-to-detector distance and other attenuation effects.

**XPS Fitting**

The Ir 4f spectra of amorphous iridium oxide were fitted using the parameters listed in **Tables S2** and **S3**.[10–15]

**Table S2**. Background fitting parameters for Ir 4f XPS spectra of amorphous iridium oxide.

| | Background | |
|---|---|---|
| | Low | High |
| Offset | -7 | 0 |
| Slope | 0.005 | 0.15 |
| Parabola | 0 | Locked |
| Psuedo Tougaard | 0 | Locked |
| Shirley step height | 1.00E-06 | 3 |

**Table S3**. Peak fitting template for amorphous iridium oxide Ir 4f XPS at B07 C at 1070 eV photon energy, 0.1 mm exit slits, 20 eV pass energy. To improve the quality of the fit, if initial fits show an $Ir^{3+}$ or $Ir^{5+}$ (peaks 4 and 5) area below 0.1 the peak may be determined to not be present so be removed from the model or locked into 0.001 peak area.

| | Peak 1 $Ir^{4+}$ | | Peak 2 $Ir^{4+}$ satellite 1 | | Peak 3 $Ir^{4+}$ satellite 2 | | Peak 4 $Ir^{5+}$ | | Peak 5 $Ir^{3+}$ | |
|---|---|---|---|---|---|---|---|---|---|---|
| Low/high limit | Low | High | Low | High | Low | High | Low | High | Low | High |
| Area | 1 | 50 | A1×0.14 | A1×0.14 | A1×0.19 | A1×0.19 | 0.001 | 50 | 0.001 | 50 |
| Position | 61.9 | 62.15 | P1 + 0.928 | P1 + 0.928 | P1 + 2.634 | P1 + 2.634 | P3 + 0.88 | P3 + 0.88 | P1 - 0.834 | P1 - 0.834 |
| Width | 1.618 | Locked | 1.762 | Locked | 3.385 | Locked | 3.566 | Locked | 0.697 | Locked |
| G:L | 0.526 | Locked | 0.526 | Locked | 0.526 | Locked | 0.526 | Locked | 0.526 | Locked |
| Asymmetry | 0.893 | Locked | 0.001 | Locked | 0.529 | Locked | 0.373 | Locked | 0.001 | Locked |
| Asymmetry translation | 0.001 | Locked | 0.001 | Locked | 0.001 | Locked | 0.001 | Locked | 0.001 | Locked |
| Doublet ratio | 0.75 | Locked | 0.75 | Locked | 0.75 | Locked | 0.75 | Locked | 0.75 | Locked |
| Doublet shift | 3 | Locked | 3 | Locked | 3 | Locked | 3 | Locked | 3 | Locked |
| Doublet broadening | 1.029 | Locked | 1.029 | Locked | 1.029 | Locked | 1.029 | Locked | 1.029 | Locked |

To fit O 1s XPS spectra of the amorphous iridium oxide the parameters set out in **Table S4** and **S5** are used.[16–20]

**Table S4**. Background fitting parameters for O 1s spectra of iridium oxide.

| | Background | |
|---|---|---|
| | Low | High |
| Offset | -28.717 | 0 |
| Slope | 0.0001 | 0.0732 |
| Parabola | 0 | Locked |
| Psuedo Tougaard | 0 | Locked |
| Shirley step height | 1.00E-06 | 0.5 |

**Table S5**. Peak fitting template for iridium oxides O 1s XPS at B07 C at 1540 eV photon energy, 0.05 mm exit slits, 20 eV pass energy If peak 5 has negligible area (<0.05) the peak is removed from the fitting model for the spectrum to minimise the number of components to fit.

| | Peak 1 Oxide | | Peak 2 Hydroxide | | Peak 3 Liquid water | | Peak 4 Water vapour | | Peak 5 529(.2) | |
|---|---|---|---|---|---|---|---|---|---|---|
| Low/high limit | Low | High | Low | High | Low | High | Low | High | Low | High |
| Area | 0.01 | 10 | A1×1.25 | 10 | 0.05 | 20 | 0.05 | 20 | 0.000001 | 0.2 |
| Position | P2 – 1.56 | P2 – 1.56 | 531.4 | 531.7 | P2 + 1.39 | P2 + 1.39 | 533.38 | 536.5 | P2 – 2.4 | P2 – 2.4 |
| Width | 1.287 | Locked | 2.7 | Locked | 1.831 | Locked | 0.4 | 2 | 0.981 | Locked |
| G:L | 0.3 | Locked | 0.3 | Locked | 0.3 | Locked | 0.3 | Locked | 0.3 | Locked |
| Asymmetry | 0.001 | Locked | 0.206 | Locked | 0.093435 | Locked | 0.001 | Locked | 0.001 | Locked |
| Asymmetry translation | 0.001 | Locked | 0.001 | Locked | 0.001 | Locked | 0.001 | Locked | 0.001 | Locked |

### NEXAFS Pre-edge Fitting

To fit the O K pre-edge NEXAFS data of amorphous iridium oxide a 4 peak fitting model has been developed with 4 peaks. The first 3 peaks correspond to the $\mu_1$-O, $\mu_2$-O, and $\mu_3$-O peaks. To fit the contribution from $e_g$ orbital contributions and possible Nafion contributions, a peak has been introduced to fit these additional components and minimise their effect on the fits produced. These are limited to a peak position above 530.4 eV to ensure they are not fitting the $\mu_3$-O signal. The parameters used are set out in **Table S6** and **S7**.[16,21–27]

**Table S6**. Background fitting parameters for O K pre-edge NEXAFS of iridium oxide.

| | Background | |
|---|---|---|
| | Low | High |
| Offset | -5 | 0.999 |
| Slope | $10^{-6}$ | 0.2 |
| Parabola | 0 | Locked |
| Psuedo Tougaard | 0 | Locked |
| Shirley step height | 0 | Locked |

**Table S7**. Peak fitting template for amorphous iridium oxide O K pre-edge NEXAFS at B07 C using 0.1 mm exit slits.

| | Peak 1<br>$\mu_1$-O | | Peak 2<br>$\mu_2$-O | | Peak 3<br>$\mu_3$-O | | Peak 4<br>Additional components | |
|---|---|---|---|---|---|---|---|---|
| Low/high limit | Low | High | Low | High | Low | High | Low | High |
| Area | $10^{-6}$ | 0.5 | $10^{-6}$ | 0.5 | $10^{-6}$ | 0.5 | $10^{-6}$ | 0.5 |
| Position | 528.5 | Locked | 529 | Locked | 529.86 | Locked | 530.4 | 532 |
| Width | 0.68 | Locked | 0.926 | Locked | 1.165 | Locked | 2 | Locked |
| G:L | 0.3 | Locked | 0.3 | Locked | 0.3 | Locked | 0.3 | Locked |
| Asymmetry | 0.001 | Locked | 0.206 | Locked | 0.093435 | Locked | 0.001 | Locked |
| Asymmetry translation | 0.001 | Locked | 0.001 | Locked | 0.001 | Locked | 0.001 | Locked |

## Supplementary Figures

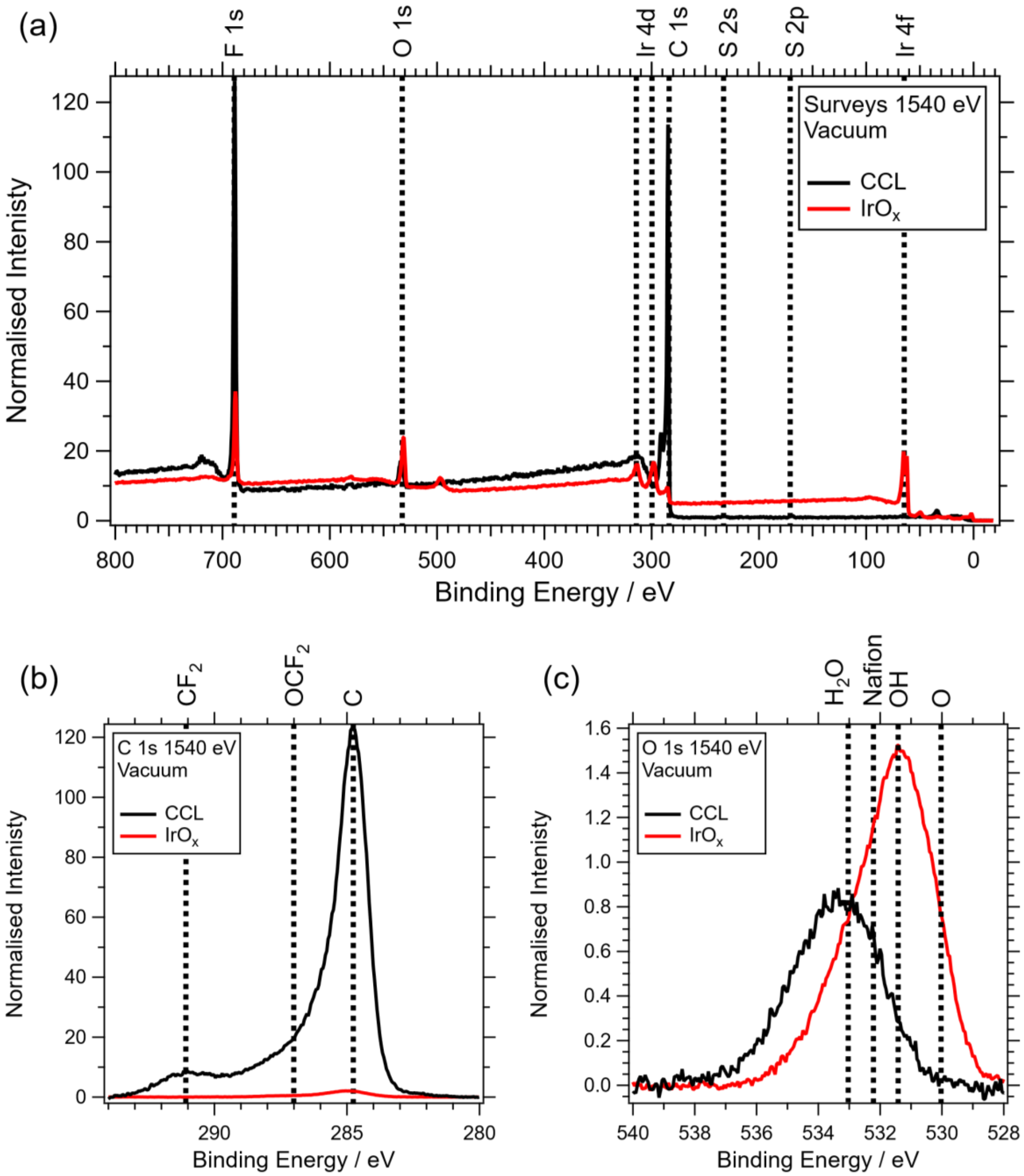


**Figure S1**. XPS of carbon conductive layer (CCL) on Nafion 115 and amorphous $IrO_x$ coated CCL O 1s under vacuum (a) surveys (b) C 1s, and (c) O 1s.

The high Nafion content of the CCL compared to the $IrO_x$ coated sample leads to significant signal from the Nafion, as seen from the F 1s contribution in the survey spectra (**Figure S1a**), the $CF_2$ and $OCF_2$ in the C 1s spectra (**Figure S1b**). When observing the O 1s region (**Figure S1c**), which is of particular interest for studies of $IrO_x$, there is negligible contribution to the signal from the $IrO_x$ coated sample, which is dominated by O and OH species on the $IrO_x$.

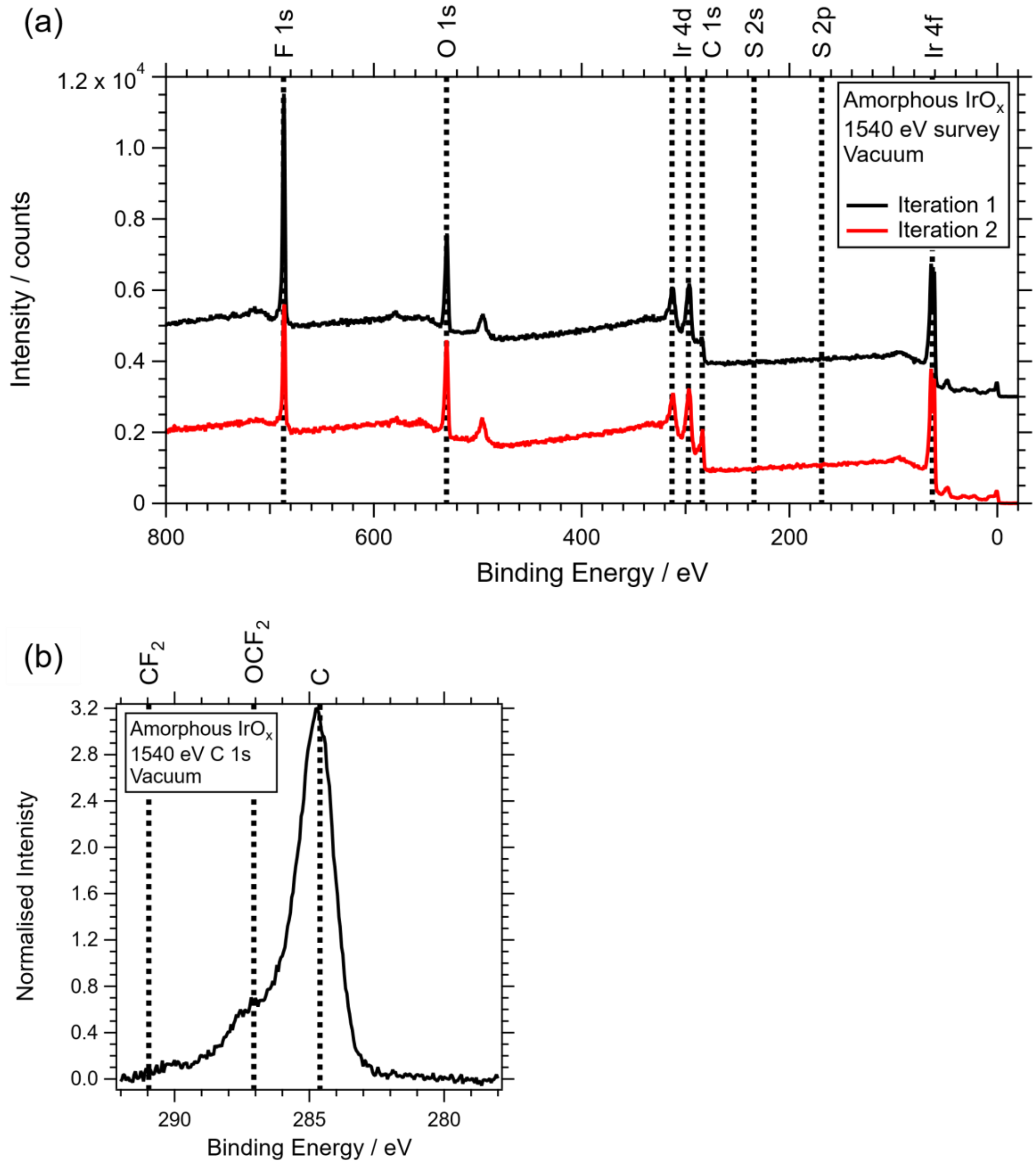


**Figure S2**. XPS of amorphous iridium oxide under vacuum conditions (a) XPS survey, (b) C 1s.

**Table S8**. Peaks observed in the survey spectra, Figure S2 (a)

| Region | Binding energy / eV |
|---|---|
| Valence band | 0 – 10 |
| Ir 4f 7/2, 5/2 | 62, 65 |
| C 1s | 284.5 |
| Ir 4d 5/2, 3/2 | 297, 312 |
| Ir 4p 3/2 | 496.5 |
| O 1s | 530 |
| F 1s | 686 |

The F 1s intensity decreases with exposure, as shown between iteration 1 and 2 as shown in **Figure S2 (a)**. The survey shows signals from iridium, carbon, oxygen and fluorine, the peaks observed are set out in **Table S1**. The lack of a peak from the S 2p region, approximately BE 169 eV, indicates the $SO_3$ species is experiencing beam damage, therefore is not detected. The C 1s spectra, **Figure S2 (b)**, consists of several features adventitious carbon BE 284.5 eV, CF BE 287 eV, $OCF_2$ BE 290 eV, with a strong feature from Ir 4d 5/2 at the end of the spectrum. The lack of $CF_2$ BE 293 eV and $CF_3$ BE 295 eV features may be symptomatic of beam damage but also obscured by the Ir 4d 5/2 peak.[15,28] There is clear beam damage in the F 1s region, **Figure S3**, with the intensity reducing as a function of time irradiated. The peak position shifts to higher binding energy and in FWHM it reduces. This indicates there is a change in the chemical environments of the Nafion and a loss of Nafion on the surface.

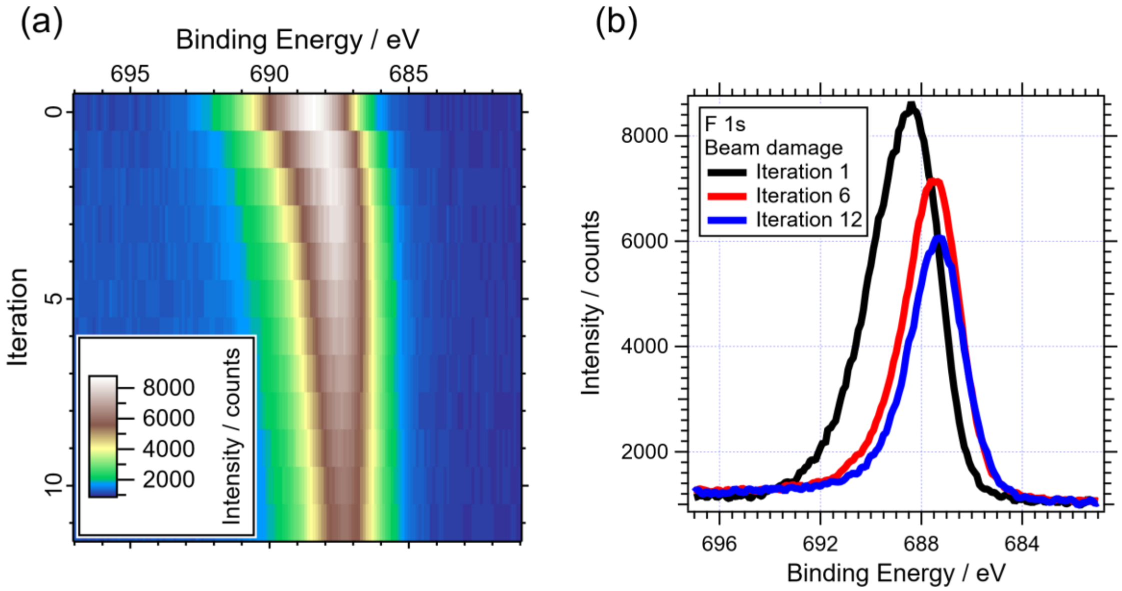


**Figure S3.** Vacuum F 1s XPS of a pristine region showing (a) image plot of F 1s XPS showing all iterations, (b) plot of selected F 1s iterations.

When electrolyte flow and water vapor is introduced into the chamber there is a significant change in the XPS. **Figure S4 (a)** XPS survey shows contributions from iridium and oxygen, **Table S8**. The lack of strong C 1s, F 1s peaks, or a S 2p peak suggests, when in the presence of water, the Nafion XPS signal is low. The C 1s spectrum, **Figure S4 (b)**, has very low intensity and is dwarfed by the Ir $4d^{5/2}$ peak. Therefore, for in situ spectroscopy the Nafion contribution will be deemed to be negligible. The low C 1s peak intensity suggests good iridium coverage, with the lack of a strong adventitious carbon peak from the carbon black conductive layer underneath.

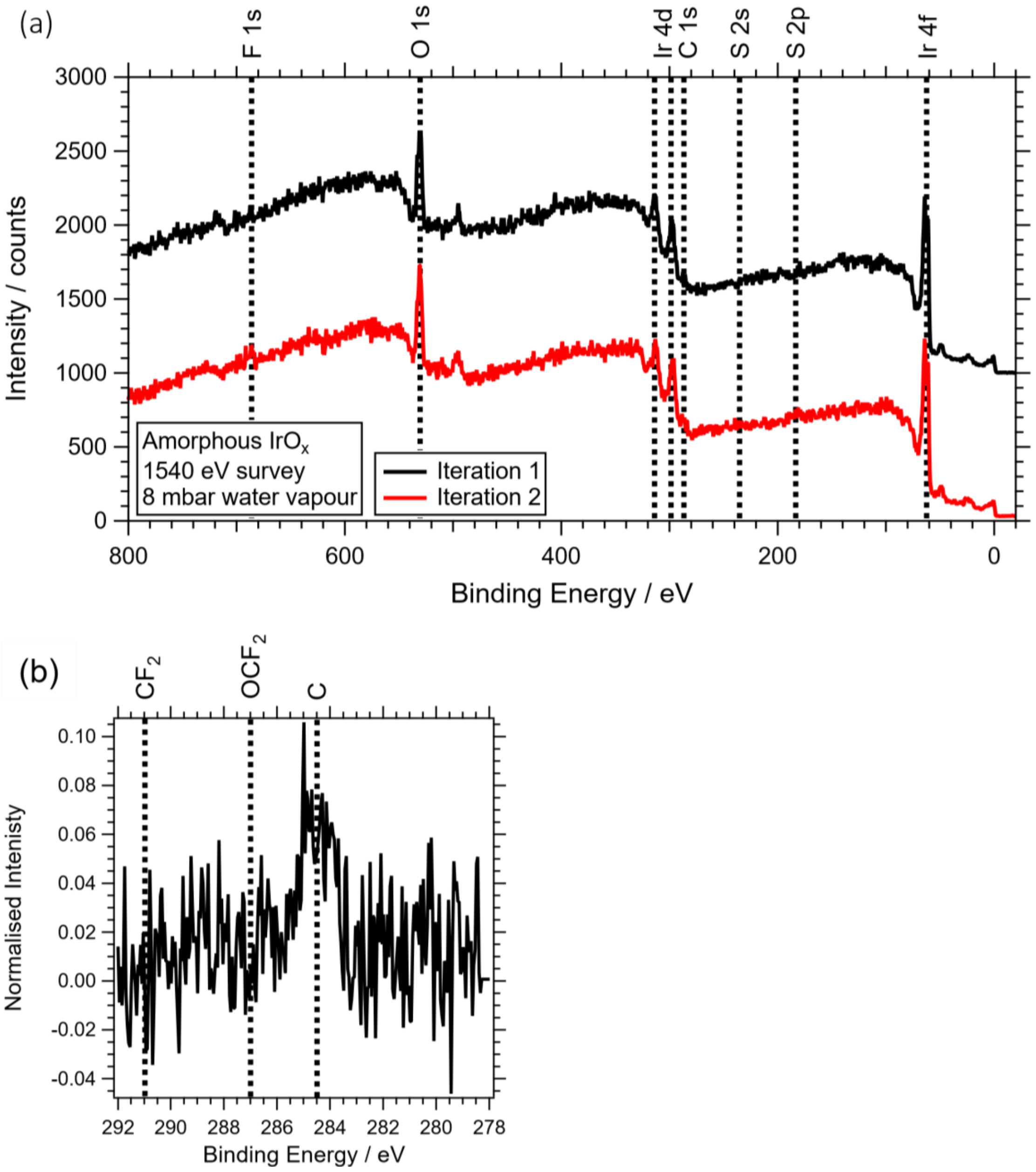


**Figure S4**. XPS of amorphous iridium oxide with electrolyte flow through the cell with 8 mbar water vapor pressure (a) XPS survey, (b) C 1s.

**Table S9**. Peaks observed in the XPS survey spectra XPS of amorphous iridium oxide, **Figure S4 (a)** with electrolyte flow through the cell with 8 mbar water vapor pressure.

| Region | Binding energy / eV |
|---|---|
| Valence band | 0 – 10 |
| Ir 4f 7/2, 5/2 | 62, 65 |
| Ir 4d 5/2, 3/2 | 297, 312 |
| Ir 4p 3/2 | 496.5 |
| O 1s | 530 |

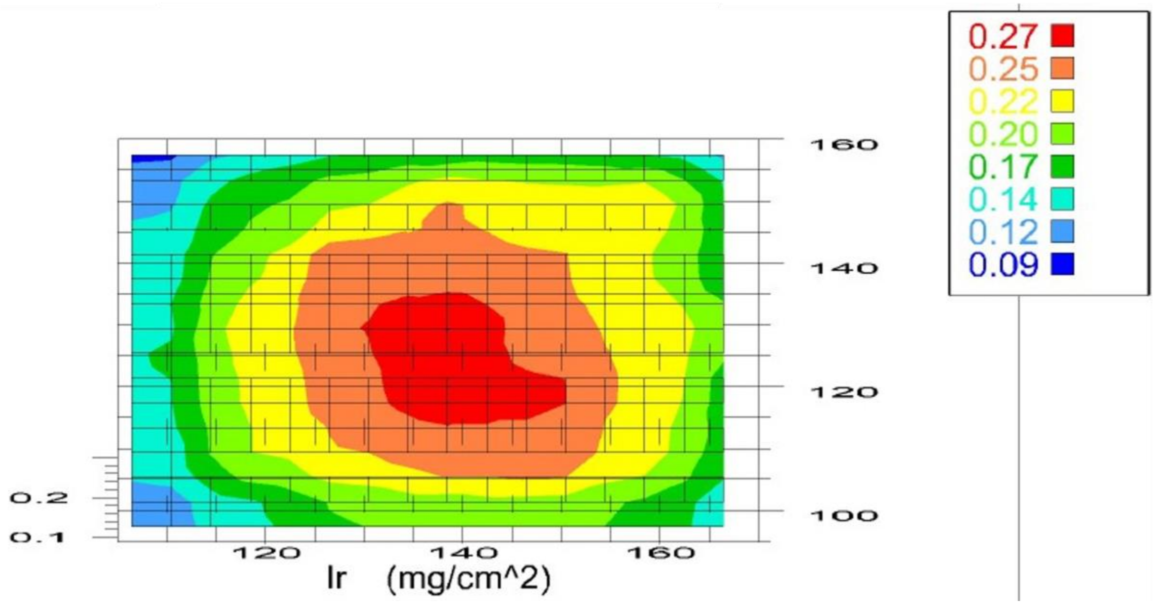


**Figure S5.** Heatmap of amorphous iridium oxide on carbon on Nafion 115 loading in 1 cm intervals across the 7 X 7 cm sheet.

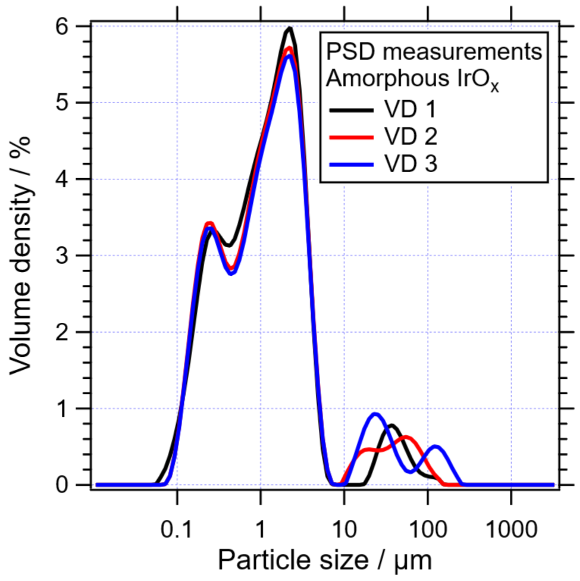


**Figure S6**. PSD measurement of processed amorphous $IrO_x$ prior to spray coating showing volume density (VD) vs particle size (on a logarithmic x-axis).

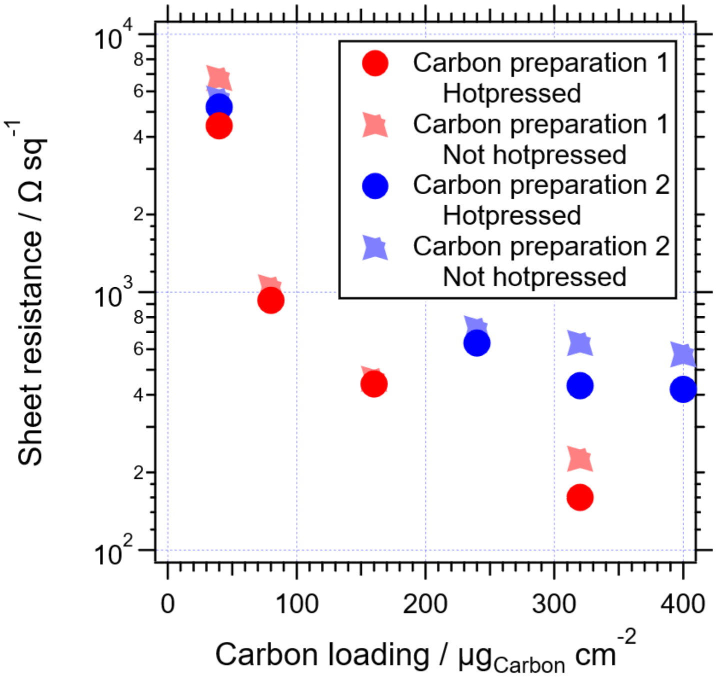


**Figure S7.** Plot of sheet resistance measured using 4-point probe conductivity against carbon thickness presenting the same data as **Figure 3(a)** with a log-scaled y-axis. Carbon preparation series 1 and 2 displayed before and after hot pressing.

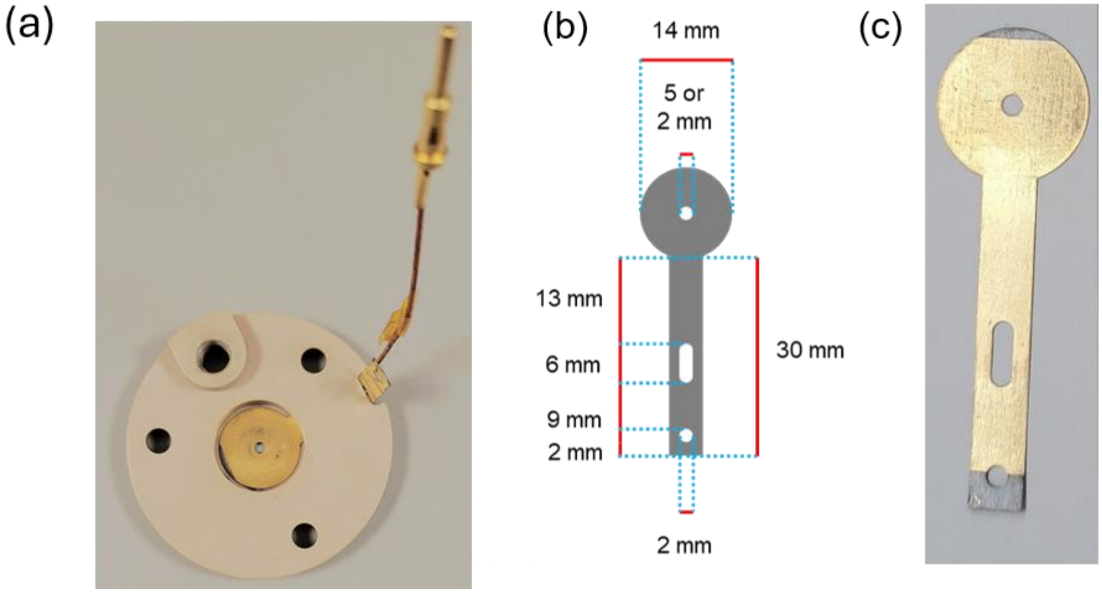


**Figure S8.** (a) Au coated Ta current contact for WEA installed on the lid that seal the flow cell for in situ XPS measurements, (b) Schematic diagram of contact, and (c) photograph of Au coated Ta contact.

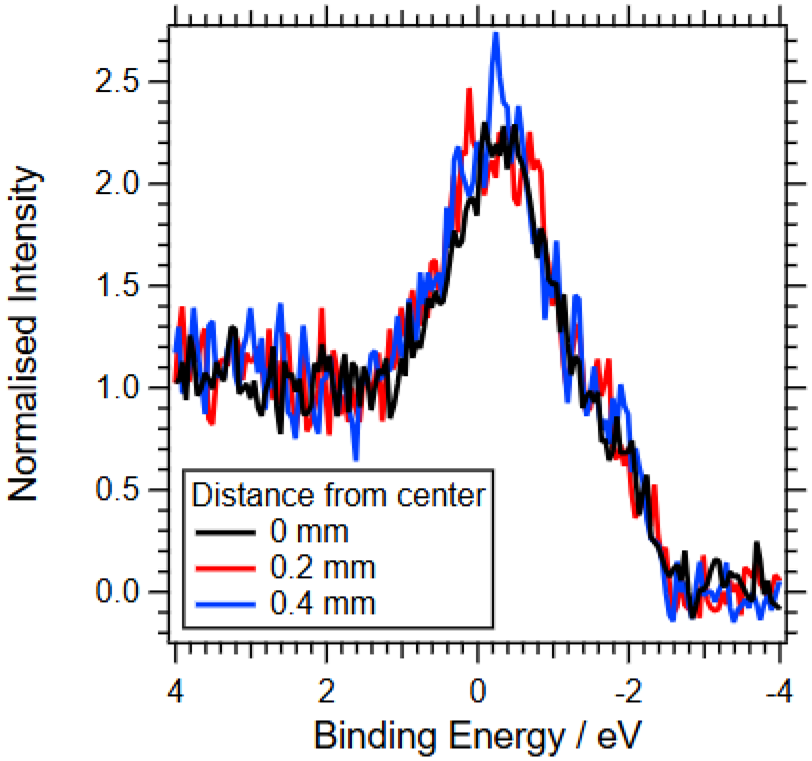


**Figure S9.** Uncalibrated and normalized in situ valence-band XPS spectra acquired at three lateral positions along a single line across a crystalline $IrO_2$ electrode. Measurements were performed at 1070 eV photon energy in 8 mbar $H_2O$ vapor while holding the electrode at 1.6 V, resulting in a current of ~0.25 mA. No charge compensation was applied. The spectra show no measurable shift in the valence-band position, indicating negligible lateral potential gradients across the probed region.

The potential drop between the edge and the centre of a circular electrode can be estimated using the eqn S3: [29]

$$\Delta V = \frac{R_S I \ln 2}{\pi} \quad \textbf{S3}$$

where ΔV is the voltage drop from the edge to the centre, $R_S$ is the sheet resistance of the conductive underlayer and I is the total current. For the present system, x is 2 mm. For a current density of 10 mA $cm^{-2}$ with a 2 mm aperture, the estimated potential drop across the sample is ~11 mV relative to the applied potential of 1.6 V (crystalline sample). Notably, the maximum ΔV occurs at the centre of the aperture, which represents the worst-case position; measurements performed near the aperture edge (at least ≈ 0.5 mm from the centre) experience even smaller potential variations due to efficient electron distribution through the carbon underlayer. This confirms that the WEA design maintains uniform electrochemical

conditions across the illuminated catalyst area, ensuring reliable XPS and NEXAFS measurements.

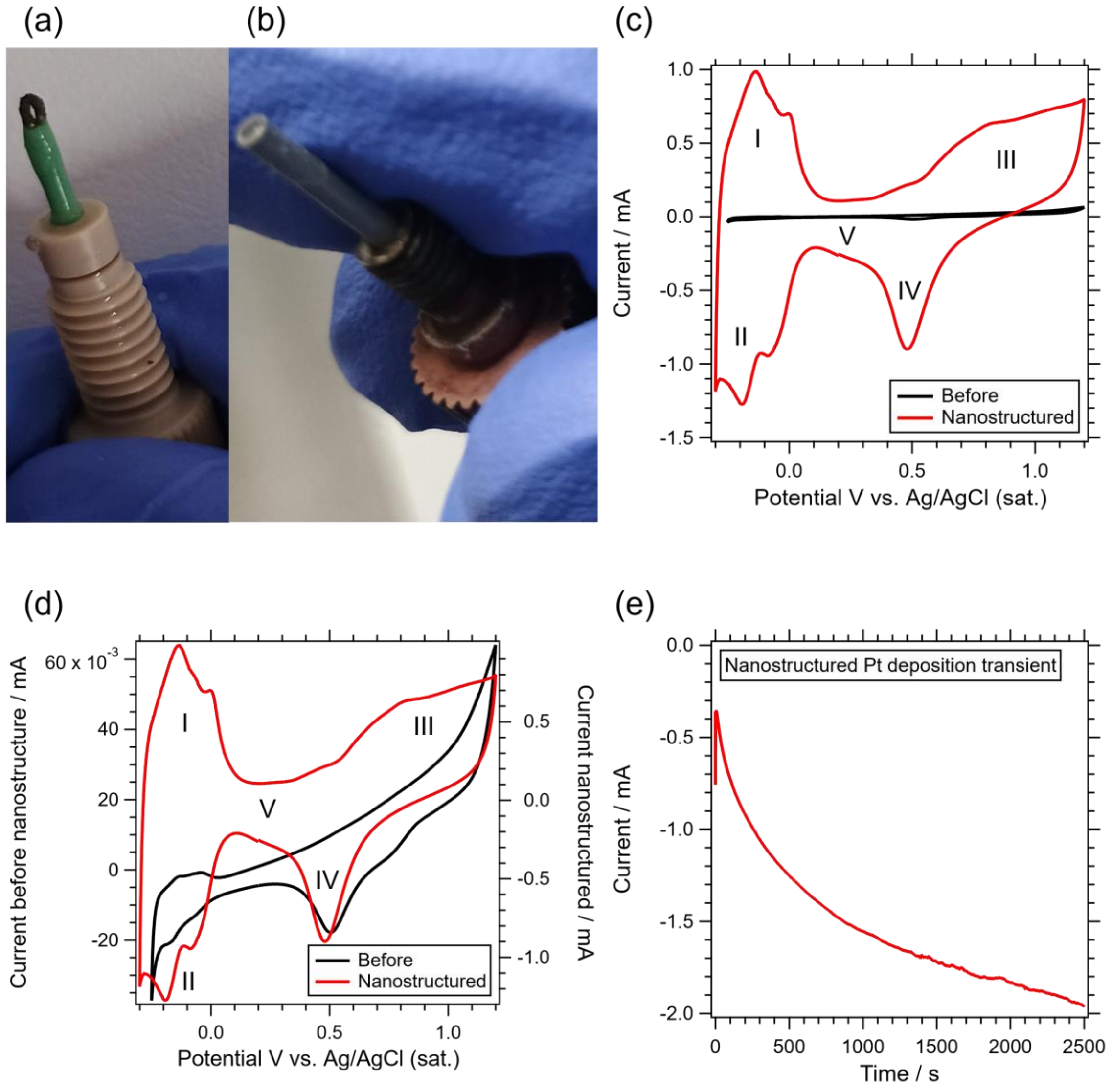


**Figure S10.** Counter electrode photographs (a) initial looped Pt wire, (b) larger Pt wire. (c, d) Cyclic voltammograms of counter electrode before and after deposition of nanostructured; (c) both traces on the same y-axis, and (d) dual y-axis chart to show differences in cyclic voltammetry. (e) Deposition transient of Pt electrode.

**Table S10.** Features in cyclic voltammograms of Pt electrodes in Figure S10. [4]

| Feature annotation | Potential vs Ag/AgCl (sat.) | Feature |
|---|---|---|
| I | -0.3 V to 0.1 V oxidatative | H adsorption |
| II | 0.1 to -0.3 V reductive | H adsorption |
| III | 0.6 V to 1.2 V oxidative | $Pt^0$ à PtO |
| IV | 0.5 V reductive | PtO à $Pt^0$ |
| V | 0.1 V to 0.4 V | Double layer capacitance |

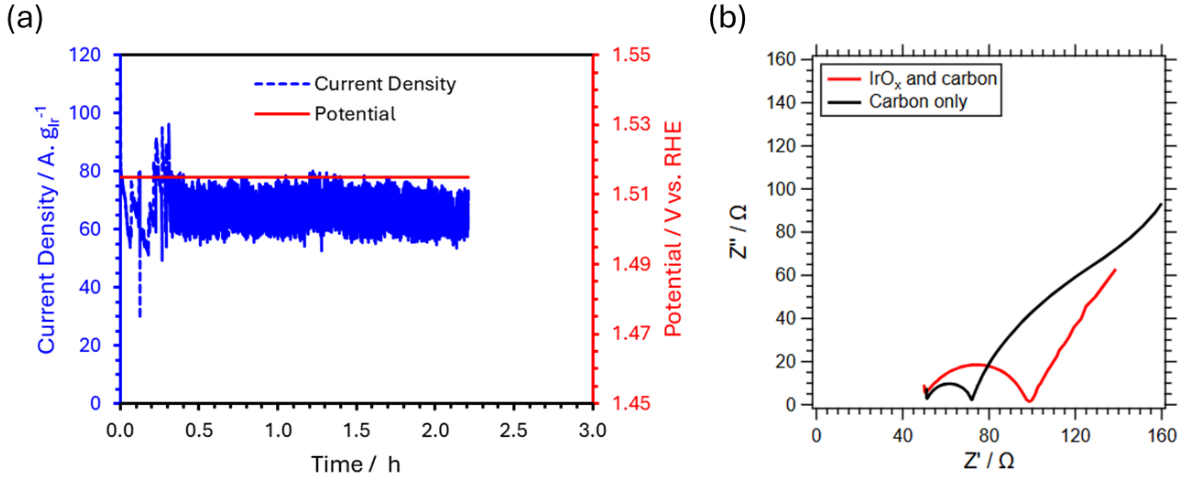


**Figure S11**. (a) Dual-axis plot of current density (left axis) and potential (right axis) as a function of time under in situ conditions. (b) Nyquist plots of the WEA with and without $IrO_x$ at 0.8 V; the data were recorded on the bench under ambient condition with the same spectro-electrochemical flow cell that was used for the beamline experiments. 0.1 M $H_2SO_4$ electrolyte flowing continuously at 2 mL min$^{-1}$.

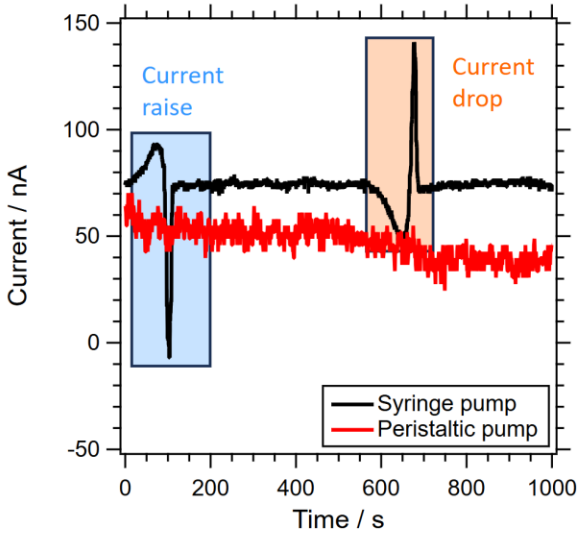


**Figure S12.** Current vs time traces of in situ electrochemistry comparing electrolyte flow using syringe pump and the peristaltic pump. The example data presented here were collected on crystalline $IrO_2$ during a potential hold at 1.1 V.

## Water doser

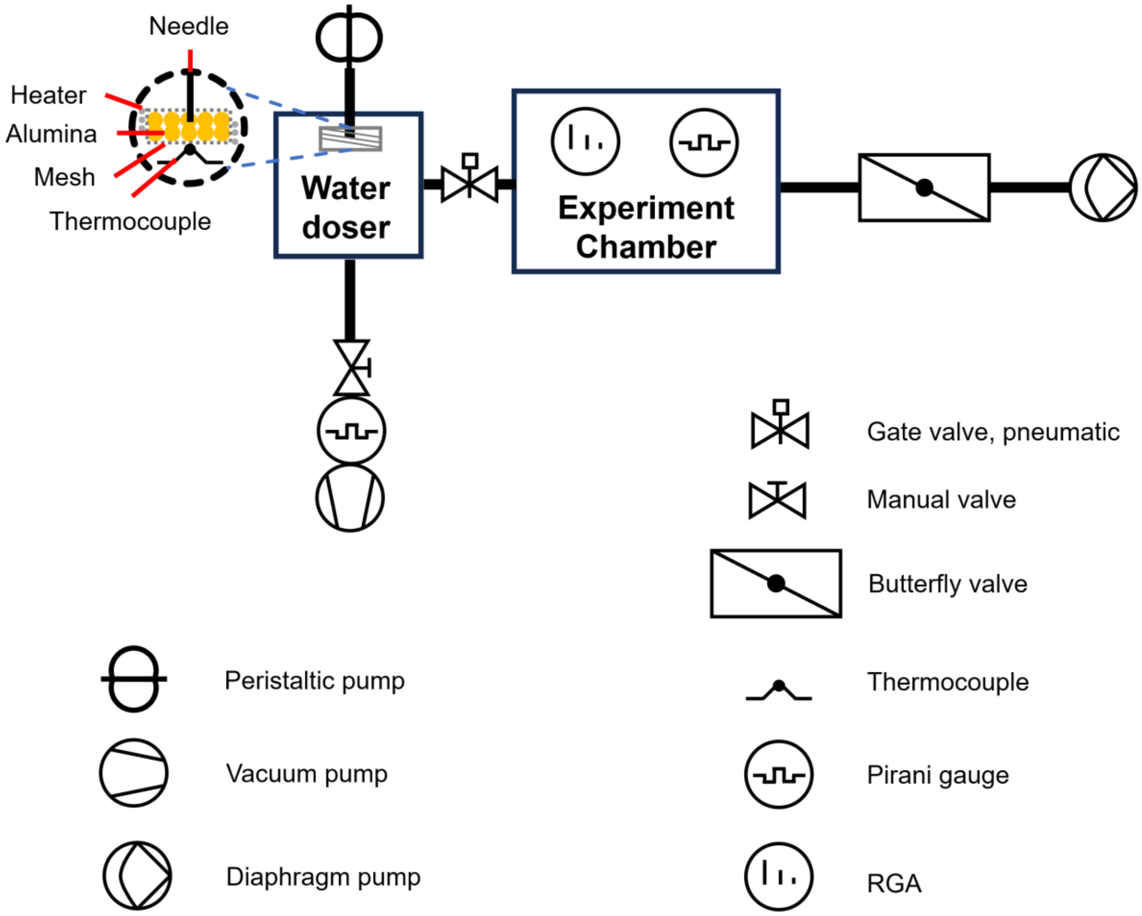


**Figure S13.** A schematic showing a new water doser technique developed at the B07 beamline, which was used in conjunction with a PID-controlled butterfly valve pumping system to achieve better pressure control within the experimental chamber for *in situ* XPS/NEXAFS measurements. In this setup, a peristaltic pump delivers water into a needle positioned in an alumina bed, enclosed within a metal mesh that has a thermocouple spot-welded to it. A pneumatically controlled valve regulates the introduction of water vapor into the experimental chamber, while a butterfly valve, controlled by the PID system, adjusts the pumping rate to maintain stable pressure in the near-ambient pressure range.

**Water coverage derivations**: From the fitting data the fraction of signal from iridium oxide (O and OH signal) can be compared to that of the water. The water layer forms on top of the iridium oxide. Eqn S4 and S5 were used to calculate the thickness of the water layer from the signal obtained.

$$I_{IrO_x} = I0_{IrO_x} \times e^{-\frac{t}{\cos\alpha \times \lambda_{IrO_x}}} \quad \text{S4}$$

$$I_{H_2O} = I0_{H_2O}\left(1 - e^{-\frac{t}{\cos\alpha \times \lambda_{H_2O}}}\right) \quad \text{S5}$$

Where $I_{IrO_x}$ is the signal intensity from the iridium oxide. $I_{H_2O}$ is the signal intensity from the water film. $I0_{IrO_x}$ is the signal intensity from an infinitely thick film of iridium oxide. $I0_{H_2O}$ is the signal intensity from an infinitely thick film of water. $t$ is the thickness of the water layer. $\lambda_{IrO_x}$ is the inelastic mean free path (IMFP) of the $IrO_x$ photoelectrons through the $H_2O$ (liq) layer. $\lambda_{H_2O}$ is the IMFP of the $H_2O$ photoelectrons through the $H_2O$ (liq) layer. Using the TPP-2M equation [30,31] the IMFP at 1008.5 eV (OH species) and 1007 eV ($H_2O$ (liq)) are 29.08 and 29.04 Å, since the difference between these values is negligible we approximate $\lambda_{IrO_x} = \lambda_{H_2O} = \lambda$ and assumed to be 29.08 Å. $\alpha$ is the angle of the electron analyser to the normal of the sample, which is 0° when using beamline B07, hence cos α = 1. Eqn S4 and S5 can be combined and simplified to eqn S6:

$$\frac{I_{IrO_x}}{I_{H_2O}} = \frac{I0_{IrO_x}}{I0_{H_2O}} \times \frac{e^{-\frac{t}{\cos\alpha \times \lambda_{IrO_x}}}}{1 - e^{-\frac{t}{\cos\alpha \times \lambda_{H_2O}}}} \quad \text{S6}$$

From fitting $\frac{I_{IrO_x}}{I_{H_2O}}$ can be determined experimentally. Due to variation in sample position, we are unable to measure $I0_{IrO_x}$ and $I0_{H_2O}$ reliably during the experiments without significant error due to the variation in attenuation from the water vapour contribution to the signal (**Figure S14**). However, since both species are measured by the same analyser, under the same conditions with similar kinetic energies, the $\frac{I0_{IrO_x}}{I0_{H_2O}}$ can be calculated from eqn S7:

$$\frac{\rho(O)_{IrO_x} \times \delta_O}{\rho(O)_{H_2O} \times \delta_O} = \frac{\rho(O)_{IrO_x}}{\rho(O)_{H_2O}} \quad \text{S7}$$

Where $\rho(O)_{IrO_x}$ and $\rho(O)_{H_2O}$ are the molar densities of oxygen in $IrO_x$ and $H_2O$ respectively (mol $cm^{-3}$), and $\delta_O$ is the X-ray cross section of O (equal, therefore cancelling). The molar densities can be calculated from eqn S8:

$$\rho(O) = \frac{D \times n_O}{m_r} \quad \text{S8}$$

Where $D$ is density g cm$^{-3}$. $n_O$ is the number of oxygen atoms per mole. $m_r$ is the relative formula mass, g mol$^{-1}$. For water $D$ = 0.9982 g cm$^{-3}$ (the sample temperature is typically around 21.8 °C, therefore the density of water at 20 °C is used), $n_O$ = 1, $m_r$ = 18.02 g mol$^{-1}$. [32] For $IrO_2$ $D$ = 11.7, $n_O$ = 2, $m_r$ = 224.2 g mol$^{-1}$. [33] The density of $IrO_x$ used is assumed to be equal to that of $IrO_2$.

If equation 4 is simplified to eqn S9:

$$A = B \times \frac{e^{-\frac{t}{\lambda}}}{1 - e^{-\frac{t}{\lambda}}} \quad \text{S9}$$

Where $A = \frac{I_{IrO_x}}{I_{H_2O}}$ and $B = \frac{I0_{IrO_x}}{I0_{H_2O}}$. This equation can then be solved for $t$, eqn S10-16:

$$A\left(1 - e^{-\frac{t}{\lambda}}\right) = Be^{-\frac{t}{\lambda}} \quad \text{S10}$$

$$A - Ae^{-\frac{t}{\lambda}} = Be^{-\frac{t}{\lambda}} \quad \text{S11}$$

$$A = Ae^{-\frac{t}{\lambda}} + Be^{-\frac{t}{\lambda}} \quad \text{S12}$$

$$A = e^{-\frac{t}{\lambda}}(A + B) \quad \text{S13}$$

$$\frac{A}{A + B} = e^{-\frac{t}{\lambda}} \quad \text{S14}$$

$$\ln\left(\frac{A}{A + B}\right) = -\frac{t}{\lambda} \quad \text{S15}$$

$$-\lambda \ln\left(\frac{A}{A + B}\right) = t \quad \text{S16}$$

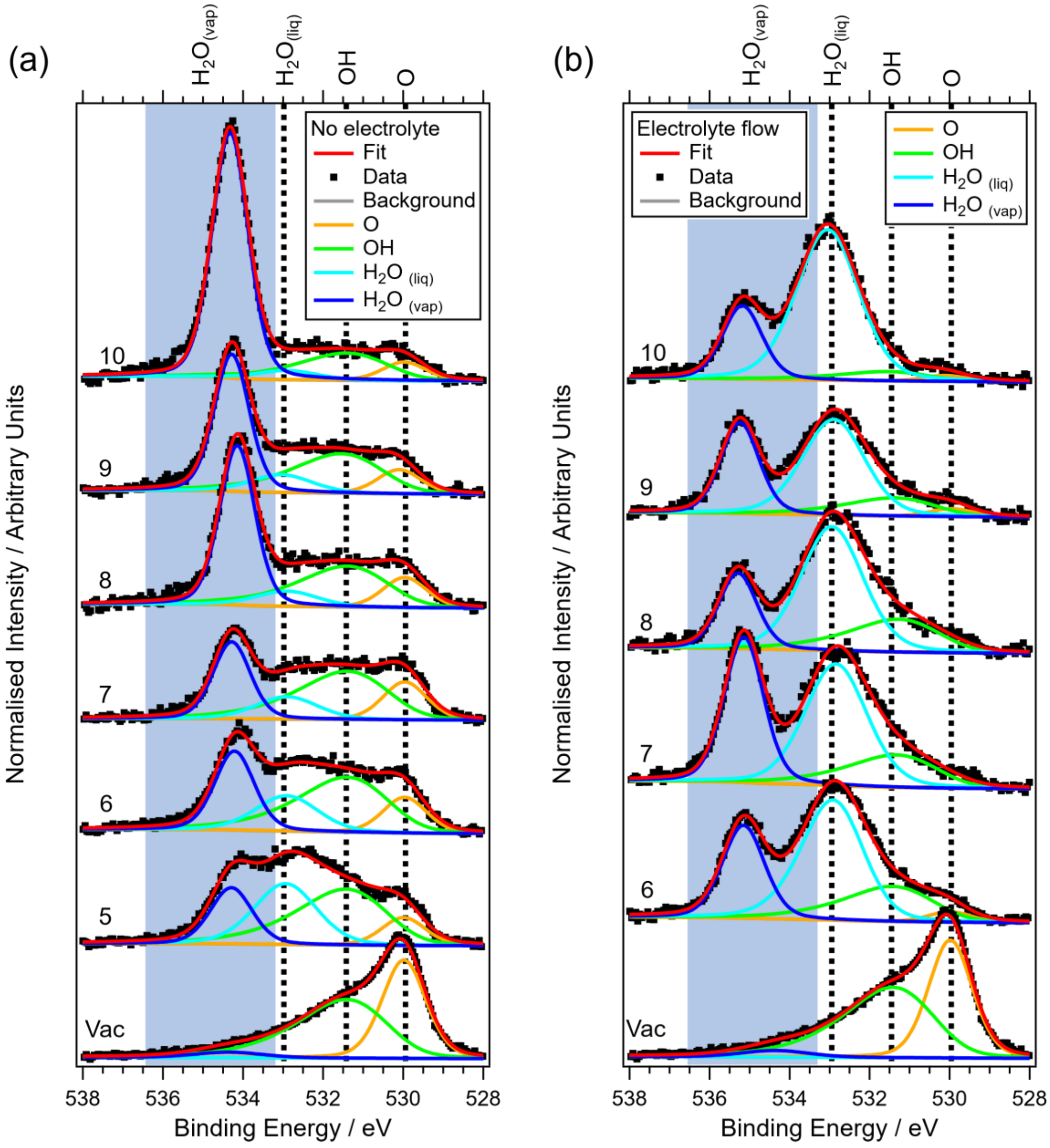


**Figure S14**. O 1s XPS of crystalline iridium oxide as a function of water vapor pressure (a) without and (b) with electrolyte in the cell.

## In situ spectroscopy results

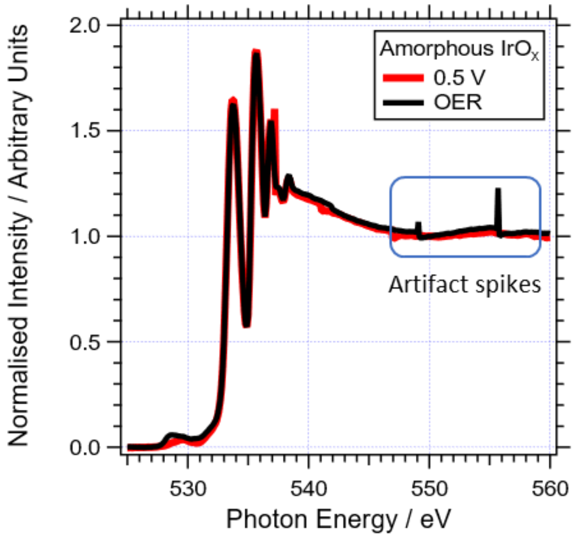


**Figure S15**. Full O K-edge NEXAFS TEY spectra recorded at 0.5 V and under OER conditions. The two spikes observed above 545 eV in the OER spectrum are random, rare artifacts and do not reflect the sample's intrinsic features.

**Table S11**. Fitting results from Ir 4f XPS showing the percentage of $Ir^{3+}$, $Ir^{4+}$, and $Ir^{5+}$ oxidation states.

| | % $Ir^{3+}$ | % $Ir^{4+}$ | % $Ir^{5+}$ |
|---|---|---|---|
| 0.5 V | 5.7 | 94.3 | 0.0 |
| OER | 0.0 | 53.1 | 46.9 |

**Table S12**. Fitting results from O 1s XPS showing the percentage of the 529.2 eV peak, O, OH, and liquid water species.

| | 529.2 eV | O | OH | $H_2O_{(liq)}$ |
|---|---|---|---|---|
| 0.5 V | 0.0 | 0.6 | 86.8 | 12.6 |
| OER | 3.4 | 10.2 | 74.2 | 12.2 |

**Table S13**. Fitting results from AEY and TEY pre-edge NEXAFS spectra showing the percentage of the $\mu_1$–O, $\mu_2$–O, and $\mu_3$–O species.

| | % $\mu_1$–O | % $\mu_2$–O | % $\mu_3$–O |
|---|---|---|---|
| 0.5 V TEY | 0.0 | 22.3 | 77.7 |
| 0.5 V AEY | 0.0 | 25.9 | 74.1 |
| OER TEY | 11.2 | 43.0 | 45.8 |
| OER AEY | 8.9 | 53.9 | 37.2 |

## References


(1) Akbar, S.; Elliott, J. M.; Rittman, M.; Squires, A. M. Facile Production of Ordered 3D Platinum Nanowire Networks with “Single Diamond” Bicontinuous Cubic Morphology. *Advanced Materials* **2013**, *25* (8), 1160–1164. https://doi.org/10.1002/adma.201203395.

(2) Barauskas, J.; Landh, T. Phase Behavior of the Phytantriol/Water System. *Langmuir* **2003**, *19* (23), 9562–9565. https://doi.org/10.1021/la0350812.

(3) Doña Rodríguez, J. M.; Herrera Melián, J. A.; Pérez Peña, J. Determination of the Real Surface Area of Pt Electrodes by Hydrogen Adsorption Using Cyclic Voltammetry. *J. Chem. Educ.* **2000**, *77* (9), 1195. https://doi.org/10.1021/ed077p1195.

(4) Biegler, T.; Rand, D. A. J.; Woods, R. Limiting Oxygen Coverage on Platinized Platinum; Relevance to Determination of Real Platinum Area by Hydrogen Adsorption. *J. Electroanal. Chem. Interfacial Electrochem.* **1971**, *29* (2), 269–277. https://doi.org/10.1016/S0022-0728(71)80089-X.

(5) Kovalensky, J.; Quinn, T. J. *International Bureau of Weights and Measures*, 9th ed.; International Bureau of Weights and Measures: Paris, 2024.

(6) Kumar, S.; Counter, J. J. C.; Grinter, D. C.; van Spronsen, M.; Ferrer-Escorihuela, P.; Large, A.; Orzech, M. W.; Jerzy Wojcik, P.; Held, G. An Electrochemical Flow Cell for Operando XPS and NEXAFS Investigation of Solid-Liquid Interfaces. *Journal of Physics: Energy* **2024**, *6*, 036001. https://doi.org/10.1088/2515-7655/ad54ee.

(7) Held, G.; Venturini, F.; Grinter, D. C.; Ferrer, P.; Arrigo, R.; Deacon, L.; Quevedo Garzon, W.; Roy, K.; Large, A.; Stephens, C.; Watts, A.; Larkin, P.; Hand, M.; Wang, H.; Pratt, L.; Mudd, J. J.; Richardson, T.; Patel, S.; Hillman, M.; Scott, S. Ambient-Pressure Endstation of the Versatile Soft X-Ray (VerSoX) Beamline at Diamond Light Source. *J. Synchrotron Radiat.* **2020**, *27* (5), 1153–1166. https://doi.org/10.1107/S1600577520009157.

(8) Tanuma, S.; Powell, C. J.; Penn, D. R. Calculations of Electron Inelastic Mean Free Paths. V. Data for 14 Organic Compounds over the 50–2000 EV Range. *Surface and Interface Analysis* **1994**, *21* (3), 165–176. https://doi.org/10.1002/SIA.740210302.

(9) Powell, C. J.; Jablonski, A. *NIST Electron Inelastic-Mean-Free-Path Database - Version 1.2*, 1.2.; National Institute of Standards and Technology: Gaithersburg, 2010. https://doi.org/10.18434/T48C78.

(10) Retuerto, M.; Pascual, L.; Torrero, J.; Salam, M. A.; Tolosana-Moranchel, Á.; Gianolio, D.; Ferrer, P.; Kayser, P.; Wilke, V.; Stiber, S.; Celorrio, V.; Mokthar, M.;

Sanchez, D. G.; Gago, A. S.; Friedrich, K. A.; Peña, M. A.; Alonso, J. A.; Rojas, S. Highly Active and Stable OER Electrocatalysts Derived from Sr2MIrO6 for Proton Exchange Membrane Water Electrolyzers. *Nat. Commun.* **2022**, *13* (1), 7935. https://doi.org/10.1038/s41467-022-35631-5.

(11) Liao, F.; Yin, K.; Ji, Y.; Zhu, W.; Fan, Z.; Li, Y.; Zhong, J.; Shao, M.; Kang, Z.; Shao, Q. Iridium Oxide Nanoribbons with Metastable Monoclinic Phase for Highly Efficient Electrocatalytic Oxygen Evolution. *Nat. Commun.* **2023**, *14* (1), 1248. https://doi.org/10.1038/s41467-023-36833-1.

(12) Sanchez Casalongue, H. G.; Ng, M. L.; Kaya, S.; Friebel, D.; Ogasawara, H.; Nilsson, A. InSitu Observation of Surface Species on Iridium Oxide Nanoparticles during the Oxygen Evolution Reaction. *Angewandte Chemie - International Edition* **2014**, *53* (28), 7169–7172. https://doi.org/10.1002/anie.201402311.

(13) Augustynski, J.; Koudelka, M.; Sanchez, J.; Conway, B. E. ESCA Study of the State of Iridium and Oxygen in Electrochemically and Thermally Formed Iridium Oxide Films. *J. Electroanal. Chem. Interfacial Electrochem.* **1984**, *160* (1–2), 233–248. https://doi.org/10.1016/S0022-0728(84)80128-X.

(14) Chen, R. S.; Chang, H. M.; Huang, Y. S.; Tsai, D. S.; Chattopadhyay, S.; Chen, K. H. Growth and Characterization of Vertically Aligned Self-Assembled IrO2 Nanotubes on Oxide Substrates. *J. Cryst. Growth* **2004**, *271* (1–2), 105–112. https://doi.org/10.1016/J.JCRYSGRO.2004.07.036.

(15) Moulder, J. F.; Stickle, W. F.; Sobol, P. E.; Bomben, K. D. *Handbook of X-Ray Photoelectron Spectroscopy: A Reference Book of Standard Spectra for Identification and Interpretation of XPS Data*; Chastain, J., Ed.; Physical Electronics Division, Perkin-Elmer Corp.: Minnesota, 1992.

(16) Pfeifer, V.; Jones, T. E.; Velasco Vélez, J. J.; Arrigo, R.; Piccinin, S.; Hävecker, M.; Knop-Gericke, A.; Schlögl, R. In Situ Observation of Reactive Oxygen Species Forming on Oxygen-Evolving Iridium Surfaces. *Chem. Sci.* **2017**, *8* (3), 2143–2149. https://doi.org/10.1039/c6sc04622c.

(17) Freakley, S. J.; Ruiz-Esquius, J.; Morgan, D. J. The X-ray Photoelectron Spectra of Ir, IrO2 and IrCl3 Revisited. *Surface and Interface Analysis* **2017**, *49* (8), 794–799. https://doi.org/10.1002/sia.6225.

(18) Arrigo, R.; Hävecker, M.; Schuster, M. E.; Ranjan, C.; Stotz, E.; Knop-Gericke, A.; Schlögl, R. In Situ Study of the Gas-Phase Electrolysis of Water on Platinum by NAP-XPS. *Angewandte Chemie International Edition* **2013**, *52* (44), 11660–11664. https://doi.org/10.1002/anie.201304765.

(19) Byrne, C.; Zahra, K. M.; Dhaliwal, S.; Grinter, D. C.; Roy, K.; Garzon, W. Q.; Held, G.; Thornton, G.; Walton, A. S. A Combined Laboratory and Synchrotron In-Situ Photoemission Study of the Rutile TiO2 (110)/Water Interface. *J. Phys. D Appl. Phys.* **2021**, *54* (19), 194001. https://doi.org/10.1088/1361-6463/ABDDFB.

(20) Su, H.; Yang, C.; Liu, M.; Zhang, X.; Zhou, W.; Zhang, Y.; Zheng, K.; Lian, S.; Liu, Q. Tensile Straining of Iridium Sites in Manganese Oxides for Proton-Exchange Membrane Water Electrolysers. *Nat. Commun.* **2024**, *15* (1), 95. https://doi.org/10.1038/s41467-023-44483-6.

(21) Falling, L. J.; Mom, R. V.; Sandoval Diaz, L. E.; Nakhaie, S.; Stotz, E.; Ivanov, D.; Hävecker, M.; Lunkenbein, T.; Knop-Gericke, A.; Schlögl, R.; Velasco-Vélez, J. J. Graphene-Capped Liquid Thin Films for Electrochemical Operando X-Ray Spectroscopy and Scanning Electron Microscopy. *ACS Appl. Mater. Interfaces* **2020**, *12* (33), 37680–37692. https://doi.org/10.1021/acsami.0c08379.

(22) Falling geb Frevel, L. *Potential-Driven Surface Phase Transitions on Iridium (Hydr-)Oxides and Their Relation to Electrolytic Water Splitting Vorgelegt von Master of Science*; PhD Thesis; Fritz Haber Institute: Berlin, 2020.

(23) Velasco-Vélez, J. J.; Falling, L. J.; Bernsmeier, D.; Sear, M. J.; Clark, P. C. J.; Chan, T.-S.; Stotz, E.; Hävecker, M.; Kraehnert, R.; Knop-Gericke, A.; Chuang, C.-H.; Starr, D. E.; Favaro, M.; Mom, R. V. A Comparative Study of Electrochemical Cells for *in Situ* x-Ray Spectroscopies in the Soft and Tender x-Ray Range. *J. Phys. D Appl. Phys.* **2021**, *54* (12), 124003. https://doi.org/10.1088/1361-6463/abd2ed.

(24) Velesco-Velez, J.-J.; Bernsmeier, D.; Jones, T.; Zeller, P.; Carbonio, E. A.; Chuang, C.-H.; Falling, L.; Streibel, V.; Mom, R.; Hammud, A.; Haevecker, M.; Arrigo, R.; Stotz, E.; Lunkenbein, T.; Knop-Gericke, A.; Kraehnert, R.; Schlögl, R. The Rise of the Electrochemical NAPXPS Operated in the Soft X-Ray Regime Exemplified in the Oxygen Evolution Reaction on IrOx Electrocatalysts. *Faraday Discuss.* **2022**, *236*, 103–125. https://doi.org/10.1039/d1fd00114k.

(25) Velasco-Vélez, J.-J.; Carbonio, E. A.; Chuang, C.-H.; Hsu, C.-J.; Lee, J.-F.; Arrigo, R.; Hävecker, M.; Wang, R.; Plodinec, M.; Wang, F. R.; Centeno, A.; Zurutuza, A.; Falling, L. J.; Mom, R. V.; Hofmann, S.; Schlögl, R.; Knop-Gericke, A.; Jones, T. E. Surface Electron-Hole Rich Species Active in the Electrocatalytic Water Oxidation. *J. Am. Chem. Soc.* **2021**, *143* (32), 12524–12534. https://doi.org/10.1021/jacs.1c01655.

(26) Saveleva, V. A.; Wang, L.; Teschner, D.; Jones, T.; Gago, A. S.; Friedrich, K. A.; Zafeiratos, S.; Schlögl, R.; Savinova, E. R. Operando Evidence for a Universal Oxygen Evolution Mechanism on Thermal and Electrochemical Iridium Oxides. *J. Phys. Chem. Lett.* **2018**, *9* (11), 3154–3160. https://doi.org/10.1021/acs.jpclett.8b00810.

(27) Mom, R. V.; Falling, L. J.; Kasian, O.; Algara-Siller, G.; Teschner, D.; Crabtree, R. H.; Knop-Gericke, A.; Mayrhofer, K. J. J.; Velasco-Vélez, J.-J.; Jones, T. E. Operando Structure–Activity–Stability Relationship of Iridium Oxides during the Oxygen Evolution Reaction. *ACS Catal.* **2022**, *12* (9), 5174–5184. https://doi.org/10.1021/acscatal.1c05951.

(28) Chen, C.; Levitin, G.; Hess, D. W.; Fuller, T. F. XPS Investigation of Nafion® Membrane Degradation. *J. Power Sources* **2007**, *169* (2), 288–295. https://doi.org/10.1016/J.JPOWSOUR.2007.03.037.

(29) Smits, F. M. Measurement of Sheet Resistivities with the Four-Point Probe. *Bell System Technical Journal* **1958**, *37* (3), 711–718. https://doi.org/10.1002/j.1538-7305.1958.tb03883.x.

(30) Powell, C. J.; Jablonski, A. *NIST Electron Inelastic-Mean-Free-Path Database, Version 1.2, SRD 71*, Version 1.; Gaithersburg, MD, 2010.

(31) Tanuma, S.; Powell, C. J.; Penn, D. R. Calculations of Electron Inelastic Mean Free Paths. IX. Data for 41 Elemental Solids over the 50 EV to 30 KeV Range. *Surface and Interface Analysis* **2011**, *43* (3), 689–713. https://doi.org/10.1002/sia.3522.

(32) Denwat. *Pure Water Density Standard*. https://www.sigmaaldrich.com/GB/en/product/sial/denwat?srsltid=AfmBOorSED4ssJpq8TQ_7C9hxALojukdrYzs0MDOSunkGUseJsRrU0Vm (accessed 2025-05-27).

(33) Thermo Scientific Chemicals. *Iridium(IV) oxide, Premion™, 99.99% (metals basis), Ir 84.5% min, Thermo Scientific Chemicals*.
